# LacI strikes a balance between stability and inducibility

**DOI:** 10.1093/nar/gkag296

**Published:** 2026-04-08

**Authors:** Jinwen Yuan, Malin Lüking, Spartak Zikrin, Beer Chakra Sen, Emil Marklund, David van der Spoel, David Fange, Johan Elf

**Affiliations:** Department of Cell and Molecular Biology, Science for Life Laboratory, Uppsala University, Uppsala, 75123, Sweden; Department of Cell and Molecular Biology, Science for Life Laboratory, Uppsala University, Uppsala, 75123, Sweden; Department of Cell and Molecular Biology, Science for Life Laboratory, Uppsala University, Uppsala, 75123, Sweden; Department of Cell and Molecular Biology, Science for Life Laboratory, Uppsala University, Uppsala, 75123, Sweden; Department of Biochemistry and Biophysics, Science for Life Laboratory, Stockholm University, Solna, 17165, Sweden; Department of Cell and Molecular Biology, Science for Life Laboratory, Uppsala University, Uppsala, 75123, Sweden; Department of Cell and Molecular Biology, Science for Life Laboratory, Uppsala University, Uppsala, 75123, Sweden; Department of Cell and Molecular Biology, Science for Life Laboratory, Uppsala University, Uppsala, 75123, Sweden

## Abstract

Transcription factors (TFs) efficiently locate their target DNA sequences by combining three-dimensional diffusion and one-dimensional sliding on nonspecific DNA. To balance rapid sliding with strong specific binding, TFs were proposed to switch between search and recognition conformations. For *Escherichia coli lac* repressor (LacI), the folding of the hinge helices has been implicated in the conformational switch. Here, we tested how mutations in the hinge region impact the search speed and binding stability. Based on molecular dynamics simulations, we selected two LacI mutants favoring either search or recognition conformation. We measured the binding kinetics of the mutants both *in vitro* on DNA microarrays with 2479 different Lac operators and *in vivo* via single-molecule experiments. We identified a mutation that enhances the specificity but reduces binding strength globally, and another mutation that makes the operator binding stronger but also reduces the specificity. However, the altered specificity impacts the search time less than expected. Instead, the major effect was impaired dissociation in response to Isopropyl β-D-1-thiogalactopyranoside (IPTG) induction for the strongly binding mutant. Together with earlier reports of affinity–inducibility trade-offs in LacI, our data support the model in which the trade-off is between binding stability and inducibility rather than between speed and binding stability.

## Graphical Abstract

**Graphical Abstract:**
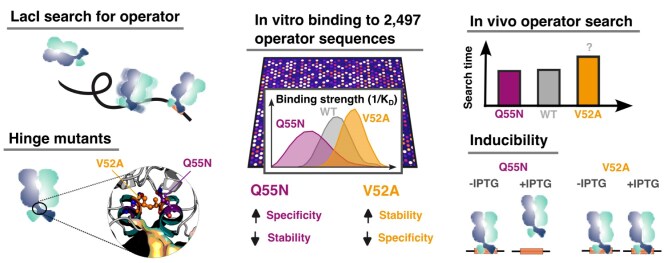

## Introduction

Transcription factors (TFs) modulate gene expression in response to environmental and developmental cues [1, 2]. They accurately and quickly bind to specific DNA sequences within a large excess of nonspecific DNA sequences in the genome. For example, the *lac* repressor (LacI) of *Escherichia coli* can find its specific target sequence among the 4.6 million genome base pairs in ∼3.5 min [3]. Fast TF search is achieved by facilitated diffusion [4], where the TF combines 3D diffusion with 1D sliding along DNA, effectively extending the target size for diffusion-limited association to the TF sliding distance [5, 6].

In the sliding state, the TF should only interact weakly (<1.5 k_B_T) with the DNA, or else the protein would be trapped at nonspecific sites [7]. At the same time, the TF should bind the specific sites strongly (>15 k_B_T), or else they will not compete well with the overwhelming number of nonspecific sites. Given a model where the TF has a static conformation, where interactions with different bases are additive, and there is a continuous distribution of binding energies, the combination of fast sliding and strong binding is not possible [7]. This is known as the speed-stability paradox. A possible resolution to the paradox was suggested by Winter *et al.* [8]. If the TF adopts two conformations, one for nonspecific interaction with DNA (search conformation), and one for specific interaction with DNA (recognition conformation), and switches between the conformations either rapidly or guided by the sequence, the speed-stability paradox can be resolved [9].

The conformation-switching model is supported by the fact that several TFs have different conformations, which are often stabilized by interactions with small molecules and other proteins. This is the case for the LacI/GalR family in bacteria, which depends on the interaction with small molecular allosteric factors [10], as well as for some human TFs, such as the ones in the Myc family, which depend on dimerization for specific DNA-binding [11]. The ability of TFs to adjust their structural and functional states in response to changes in the cellular concentrations of other molecules is crucial for their efficiency as genetic regulators [12]. Often, intrinsically disordered regions play a key role in conformational switching at specific sites for transcriptional control and signalling [13].

The question remains whether favoring the search or the recognition conformation of TFs produces predictable changes in DNA specificity, binding stability, and target search speed within living cells. Specifically, it is unclear if evolution fine-tuned the TF’s balance between the search or recognition conformation to solve the speed-stability paradox, or if there are other physical constraints that are more important. For example it has been shown that increased binding strength to the operator may come at the cost of reduced inducibility [14]. Here, we address the question for the *E. coli* TF LacI using a combination of kinetics measurements *in vitro* and *in vivo* for *in silico-*designed LacI mutants that either favor the hypothesized search or the recognition conformation.

We specifically ask if (i) a preference for the search conformation comes at the expense of lower binding probability and reduced binding strength to its operator DNA, and (ii) if a preference for the recognition conformation causes strong interactions with nonoperator DNA, making the search for the operators prohibitively slow.

Throughout the paper, binding strength, specificity, operator access time, search time, association rate and inducibility are defined as follows: (i) **binding strength**, stability, or affinity refers to the equilibrium binding constant for a DNA sequence; (ii) **specificity** refers to the difference in binding strength between an operator sequence and nonoperator sequences. A reduction in specificity means reduction in the fold change difference between operator and nonoperator sequence binding; (iii) **operator access time** refers to the time for one LacI to reach the operator sequence in the cell but not necessarily binding to it. This time includes the time spent diffusing in 3D, but also binding and sliding on nonoperator sequences that occurs before the operator is reached; (iv) **search time** is the time it takes for the *lac* operator to be bound by a LacI; (v) **association rate** is the inverse of the search time. The possibility of this direct inverse relationship in this specific case relies on the fact that searching for the stretch of DNA that contains the operator is a random nonsequential process and that the time spent nonspecifically bound to the stretch of DNA that contains the operator is negligible in relation to reaching this stretch of DNA; (vi) **inducibility** is the extent to which the fraction of operator bound LacI is reduced.

## Materials and methods

### Molecular dynamic simulations

An apo LacI dimer structure was constructed by removing DNA and ONPF from the 1EFA [15] crystal structure. Mutations to this structure were introduced using PyMOL (The PyMOL Molecular Graphics System, Schrödinger LLC) where the rotamer configurations resulting in the smallest amount of sterical clashes for each mutant were selected. The starting structures for the simulations were either the mutated (V52A, Q55N, or G58A) or the nonmutated (WT) apo LacI dimer described above. The simulations were performed with GROMACS version 2024.3 [16] and the AMBER99SB-ws [17] force field. Water molecules were modeled using TIP4P/2005 water [18]. Potassium parameters from Luo and Roux [19] were used, as standard AMBER force fields tend to overestimate the strength of nonbonded interactions for potassium [20]. We ran in the NPT ensemble at a temperature of 310 K and in a solution that contains 150 mM KCl and 5 mM magnesium ions, based on previous simulations [21]. The LINCS algorithm [22] was employed to restrain intramolecular bonds involving hydrogens. We used a 2-fs-time step for integration. Short-range interactions were calculated within 1 nm from the solute, while electrostatic interactions were calculated using the Particle–Mesh–Ewald method [23]. The stochastic velocity rescaling thermostat [24] and C-rescale barostat [25] were used for temperature and pressure coupling, respectively. The simulations which, in addition to the production runs, include 1 ns of NVT and 1 ns of NPT pre-equilibrations, were performed in five replicates for each LacI variant. In preliminary simulations, the DNA-binding domain (DBD) was observed to stick to the core domain in a manner which we deemed incompatible with nonspecific binding. To ensure that this conformation of the DBD and the core domain did not bias our helix propensity estimates, distance restraints were introduced between the DBD and the core. Here flat bottomed harmonic potentials were used. Alpha carbons on the following residues were used to add restraints: T34, E39, and D43 on the DBD, D88 on core domain on the same monomer as the selected DBD residues (i.e. T43, E39, and D43), and A106 and A110 on the core domain of the opposite monomer in the LacI dimer. Lower bounds between 1.3 and 2.2 nm, were added to the following pairs T34-A106, T34-A110, T34-D88, E34-A106, E34-A110, E34-D88, A43-A106, and A43-A110. See Supplementary Table S6 for details. Upper bounds were set to 4.2 nm for all pairs. The lower and upper bounds were set heuristically based on simulations without distance restraints.

### Quantifying the fraction of hinges that have helical conformation

**Figure 1.**
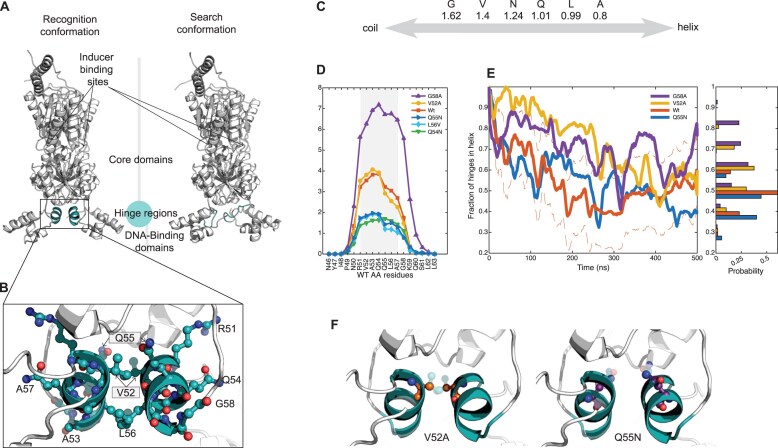
*In silico* analysis of point mutations in the LacI hinge region regarding their impact on helix propensity. (**A**) Cartoon representation of LacI dimer conformations. (Left) Recognition conformation (PDB ID: 1EFA) with hinge region highlighted in teal. (Right) Hypothetical search conformation visualized by integrating the core domain from 1EFA with the DBD from a nuclear magnetic resonance (NMR) structure (PDB ID: 1OSL) [27], as described in Lüking *et al.* [28], with hinge region similarly highlighted in teal. (**B**) Cartoon and Ball-and-Stick representation of the hinge helix residues. In the Ball-and-Stick model: Carbon atoms are shown in teal, nitrogen atoms in dark blue, and oxygen atoms in red. Residue names are labeled. (**C**) Subset of estimated characteristic energies for helix folding (excluding the formation of hydrogen bonds) in kcal mol^−1^ of different protein residues as presented by Muñoz and Serrano [29]. (**D**) Predictions of helix content per residue for short peptides in solution, where the peptide sequence contains the hinge helix and its flanking amino acids (N46-L63). Helix content per residue is predicted using empirically parameterized Agadir software [29, 30]. The part of the hinge which is in helical form in the 1EFA crystal structure is shown in gray. (**E**) *(left)* Timetraces of the fraction of hinges which have helical form (see the ‘Materials and methods’ section) in the five replicate molecular dynamics (MD) simulations for each LacI variant (solid lines). The fractions have been smoothed using a sliding window of size 10 ns. The dashed lines show 1× standard error of the mean (SEM) around the smoothed fraction of the WT for each time point. The SEM has been smoothed using a sliding window average with window size 10 ns. The RMSDs from ideal helix for residues R51–A57, used to define helical conformation as having an RMSD < 0.1 nm are shown in Supplementary Fig. S2. Averages of RMSD from each mutant shown in Supplementary Fig. S3A. *(right)* Histograms of all time-points between 300 and 500 ns for the trajectories to the left. (**F**) Cartoon and Ball-and-Stick representations of the point-mutated residues (V52A in orange and Q55N in purple) introduced into the hinge region of Wt-LacI (see point-mutation methods detailed in the ‘Materials and methods’ section). The original wild-type residues (shown half-transparent) are included for comparison and correspond to those in panel (B).

Root mean square deviations (RMSDs) from an ideal helix (Supplementary Fig. S2 which is used to generate Fig. 1E) were calculated for residues Arg51–Ala57 using the Gromacs helix function for each sampled time-point of each simulation. For each time-point the hinge region was classified as helical if the RMSD < 0.1 nm (Supplementary Fig. S2) [26]. The fraction of helical hinge regions (Fig. 1E) were calculated for each time point and for each hinge region assuming that the two hinges in the LacI dimer fold and unfold independently.

### Agadir helix predictions

The Agadir software was downloaded from https://agadir.crg.es/products#agadir. No C- or N-terminus modification, pH was set to 7, temperature was set to 300K and ionic strength was set to 200 mM.

### Significance difference test on distributions of fraction of hinge helices

**Figure 2.**
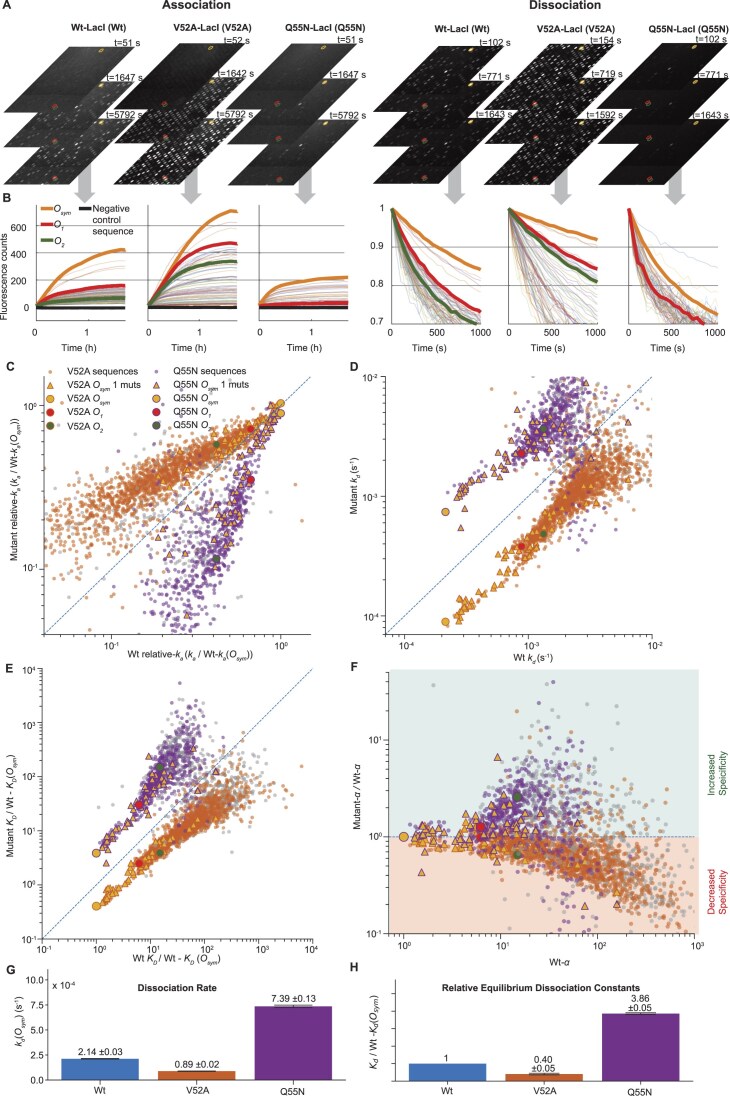
*In vitro* real-time kinetics measurement for LacI variants on protein binding microarrays (PBMs). (**A**) Representative fluorescence time-lapse images of association (*left*) and dissociation (*right*) phases for Wt-LacI (Wt) and mutant LacI variants (V52A and Q55N) on the DNA microarrays. (**B**) Real-time binding curves for O_sym_, *O_1_*, and *O_2_* operators and random 80 examples of their mutants (faint color), along with the negative control sequence. The association curves (*left*) show the increase of fluorescence intensity over time, while the dissociation curves (*right*) display the normalized fluorescence decrease from the first frame of the corresponding dissociation movie. Each curve corresponds to fluorescence intensity changes over time for a unique DNA sequence within a single PBM experiment using a specific LacI variant. The fluorescence values shown represent averaged intensities from at least three replicate DNA spots within that single PBM experiment. The curves presented here have been filtered to exclude any DNA sequences whose fitted association (*k_a_*) or dissociation (*k_d_*) rate constants showed a coefficient of variation (CV) >1; these sequences were also omitted from the calculations of the mean relative-*k_a_* and *k_d_* values displayed in panels (C) and (D). (**C**) Correlation of relative-*k_a_* values (normalized with Wt-LacI *k_a_*(*O*_sym_) value) for unique DNA sequences between Wt-LacI (x-axis) and LacI mutants (*y*-axis), with V52A (orange) and Q55N (purple) LacI variants plotted in the same panel. Sequences with high variability in relative-*k_a_* measurements (CV > 0.3) across corresponding replicates are marked in gray. Each data point represents the mean relative-*k_a_* value for a unique DNA sequence, calculated from all binding spots with the same sequence in five replicated experiments for each mutant-LacI (see individual side-by-side Wt versus Mutant PBM experiment in Supplementary Fig. S4A). (**D**) Correlation of dissociation rates, *k_d_*, between Wt (*x*-axis) and its mutants (*y*-axis), Sequences with high variability in *k_d_* measurements (CV > 0.3) are marked in gray (see individual side-by-side Wt versus Mutant PBM experiment in Supplementary Fig. S4B). (**E**) Correlation of equilibrium dissociation constants (*K*_D_) between Wt (*x*-axis) and LacI mutants (*y*-axis). *K*_D_ values, calculated from *k_d_*/*k*_a_, of each protein to different sequences are normalized with the *K*_D_ value of Wt to the *O*_sym_ operator (see individual side-by-side Wt versus Mutant PBM experiment in Supplementary Fig. S4C, and Supplementary Fig. S4D for *K*_D_ derived from 1/f_eq_, f_eq:_ fluorescence measured at/close to equilibrium. Supplementary Fig. S5 shows comparison of K_D_ values from k_d_/k_a_ and KD values from 1/f_eq_). (**F**) Specificity changes were assessed using dissociation constant (*K*_D_) estimates. For each DNA sequence (i), a LacI variant’s *K*_D_ to it was defined as the product of its *K*_D_ for the *O*_sym_ and a specificity factor, *α*(i), i.e*. K*_D_(i) = *α*(i) *K_D_*(*O*_sym_). If a mutation in LacI does not alter its DNA specificity to *O*_sym_, the *α* values for Wt-LacI and Mutant-LacI would be identical for all sequences, i.e. Wt-*α*(i) = Mutant-*α*(i) for all *i*. Normalized Mutant-*α* values (relative to Wt-*α* values) were plotted against Wt-*α* values. If a LacI mutant’s normalized α values are generally bigger than 1 (green area), that means, compared to Wt, its specificity to *O*_sym_ is increased, while values below 1 (red area) indicate that its specificity to *O*_sym_ is decreased. (**G**) Measured *k_d_*(*O*_sym_) values, shown as mean ± SEM. (**H**) *K*_D_(*O*_sym_) values are calculated from *k_d_*/*k_a_* values and normalized with Wt-*K*_D_(*O*_sym_), shown as mean ± SEM.

For each of the time traces in Fig. 2E (right), the trajectories were sampled every 15 ns starting from 300 ns. Welch’s test was carried out using the ttest2 function in MATLAB. The flag ‘vartype’ was set to ‘unequal’ to allow for different variances in the two tested distributions. For Q55N the flag ‘Tail’ was set to ‘left’ to test for the mean of Q55N being lower than Wt. For V52A and G58A the ‘Tail’ parameter was set to ‘right’ to test for the mean of the mutants being larger than Wt.

### Construction, expression, purification, and fluorophore labeling of LacI variants

The LacI-Halo constructs used in the *in vitro* DNA microarray experiments have the same LacI sequence as in our previous study [31]. Additionally, a HaloTag (underlined in sequence info in Supplementary Table S3) and a 6xHis-Tag were translationally fused to the C-terminal of LacI. LacI mutants were constructed based on the LacI-Halo construct. The V52A-LacI-Halo construct introduced an Alanine at amino acid position 52 of the LacI-Halo protein to replace Valine. The Q55N-LacI-Halo construct introduced an Asparagine at position 55 to replace the original glutamine. See sequences of LacI-Halo constructs in Supplementary Table S3.

All variants of LacI-Halo were expressed in BL21 *E. coli* cells using the pD861 plasmid, which is taken from Marklund *et al.* [32]. The resulting strains were used for overexpression of the Wt, V52A, and Q55N LacI variants, respectively. The protein expression and purification protocol, adapted from a previously published method, is described below [32]. Then, 5 ml overnight cultures were diluted into 500 ml lysogeny broth (LB) medium with 50 µg/ml kanamycin and grown at 37°C and 110 revolutions per minute (RPM) agitation. The expression of LacI was induced at OD_600_ = 0.5–1 by adding L-rhamnose to a final concentration of 0.2% (weight/volume). After 3 h, the cells were harvested by centrifugation at 6000 × *g* at room temperature for 40 min. The pellet cells were then resuspended in 25 ml BugBuster^®^ Master Mix (Merck) solution containing one ethylenediaminetetraacetic acid (EDTA)-free protease inhibitor pill (Roche), and the cell suspension was incubated on a shaking platform at 50–75 rpm for 10–20 min at room temperature. The lysate was then clarified by centrifugation at 5000 × *g* for 1 h at 4°C, followed by filtering of the supernatant through a 0.45 µm cellulose acetate membrane filter. The collected soluble extract was loaded directly onto a His GraviTrap column (Cytiva). The LacI-containing fractions were pooled and buffer-exchanged three times using 50 kDa cut-off Amicon Ultra-15 Centrifugal filters (Merck Millipore) into phosphate-buffered saline (PBS). Samples in PBS were diluted by adding an equal volume of 90% glycerol in PBS, resulting in a final storage solution of 45% glycerol in PBS. The protein was then kept at −20°C for short-term storage prior to labeling.

The labeling of LacI proteins with HaloTag^®^ Tetramethylrhodamine (TMR) Ligand (Promega) was performed in PBS + 5% glycerol. A volume of purified protein stored in 45% glycerol in PBS, as previously described, was mixed with nine volumes of PBS, and an excess amount of TMR ligand (at least a protein:ligand ratio of 1:1.1) was added to the labeling mixture. The labeling reaction mixture was incubated for 1 h at room temperature. The labeled protein was loaded onto a His GraviTrap column (Cytiva) with binding buffer (20 mM sodium phosphate, 500 mM NaCl, 20 mM imidazole), and the eluted fractions were collected using the elution buffer (20 mM sodium phosphate, 500 mM NaCl, 500 mM imidazole). The eluate was then buffer-exchanged into 1 × PBS using an Amicon^®^ Ultra Centrifugal Filter (50 kDa molecular weight cutoff). Protein concentration and labeling efficiency were determined for the TMR-labeled LacI variants using absorbance measurements at 280 nm (A_280_) and 548 nm (A_280_). The protein concentration was calculated as in Thermo Fisher Tech paper (https://documents.thermofisher.com/TFS-Assets/LSG/brochures/TR0031-Calc-FP-ratios.pdf): Protein concentration (M) = [A_280_ − (A_548_ × CF)]/ε, where ε is the protein molar extinction coefficient (81 360 M⁻¹cm⁻¹), CF (correction factor) was measured as 0.23. Labeling efficiency, expressed as moles of dye per mole of protein, was determined using: Moles dye per mole protein = A_548_/[ε' × protein concentration (M)], where ε' is the molar extinction coefficient of the TMR ligand (78 000 M⁻¹cm⁻¹). Absorbance values were averaged across at least five technical replicates. Proteins in PBS were then diluted to the desired concentration with an equal volume of 90% glycerol in PBS and were aliquoted for long-term storage at −80°C.

### Microscopy of PBMs

The design and protocols for PBMs and microscopy were replicated from a prior study [31] with the addition that the LacI variants could be flowed into different PBM-containing flow chambers on the same device (Supplementary Fig. S6), and thus, the kinetics of different variants could be measured simultaneously.

To prepare the single-stranded oligonucleotide microarrays synthesized by Agilent Technologies as PBMs, they were first synthesized into double-stranded DNA using a reaction mixture containing Cy5-dCTP according to previously published methods [31, 33]. Prior to the association and dissociation experiments, PBMs were incubated with imaging buffer (potassium phosphate 10 mM, EDTA 0.1 mM, glycerol 5%, sodium chloride 20 mM, 1 mM 2-mercaptoethanol, 0.5 mg/ml bovine serum albumin, 2% nonfat milk (Semper), final pH were adjusted with 1M potassium hydroxide to 7.2) for 1 h at room temperature (∼21°C). After incubation, association experiments were started by flowing 1.85 nM LacI dimer and 1.85 nM mutant-LacI dimer in imaging buffer into different flow cells at 450 µl/min. Four to five hours after the start of the association experiments, the dissociation experiments were started by flowing in the imaging buffer without LacI proteins at 1 ml/min.

### Microscopy and imaging

The microscope setup is the same as described in our previous study [31]. Two flow cells were imaged together in one round of imaging, each location on these two PBMs was imaged every 51 s through the TMR channel for the first 60 time points of association experiments, followed by 5-min intervals thereafter. In dissociation experiments, imaging was conducted every 51 s for the first 36 scans, then shifted to 5-min intervals.

### Analysis of reaction kinetics from PBM measurements

#### Image analysis

This image analysis workflow is designed to accurately quantify TMR fluorescence intensities from PBM images, while addressing spatial heterogeneities observed in binding patterns. These patterns are shown as elevated fluorescence at the front-flow-facing region of DNA spots, especially DNA spots that show strong affinity to the flowed LacI variant. We hypothesized that this effect resulted from the depletion of free LacI downstream of the flow front near each spot. To accurately quantify spot binding intensities unaffected by this phenomenon, we refined our previously established image analysis method [31] as described below.

Initially, following our earlier approach, merged flow-cell frames—each consisting of around 40 tiled images—were stitched together. The initial binary masks for DNA spots (*maskIni*) and their local backgrounds (*bkgMaskIni*) for an array in one PBM experiment were generated using known spot centroids and derived spot radius (*spotRadius = rowSpacing/3.9*) from Agilent’s microarray geometry specifications, which provides information including *rowSpacing*. For each DNA spot, its initial fluorescence intensity was computed by the difference between its mean spot intensity value and the corresponding local background mean intensity. Invalid spots, which are located within damaged regions (e.g. scratches, air bubbles, or tubing coverage), were manually masked using Fiji software and were excluded from further analysis.

Next, to refine spot segmentation further, DNA spots exhibiting higher initial fluorescence intensity than those corresponding to the *O_1_* operator in the first dissociation experiment frame (*im1stDissoc*) were selected. These high-affinity spots were selected because, first, they are prone to show inhomogeneous binding during early association and, secondly, they can be used to improve the localization of DNA spots. A more precise spot localization was needed because accessing the flow-front-facing region of spots requires a precise on-edge localization of whole DNA spots.

Pixels in the *im1stDissoc* image that are farther than twice the spot radius from high-affinity spot centers were replaced with local median background values, creating a refined image. Bright circular spots in this refined image were detected using MATLAB’s function imfindcircles. An affine transformation matrix that aligns the centroids of these spots with the paired spots’ centroids in *maskIni* was computed using MATLAB’s function fitgeotrans. The *maskIni* was updated, as *maskTemp*, by moving all spot centroids as specified by the inverse of the transformation matrix and expanding them slightly (1.1 × spotRadius). A separate background mask (*bkgMask*) was similarly transformed from *bkgMaskIni*.

High-affinity DNA spots, now accurately segmented with *maskTemp*, were then further processed for identifying the top 20% fluorescence intensity regions within each spot at an early association time point (t = 1650 s), where the inhomogeneous binding pattern, by visual inspection, were evaluated to be the most prominent. The spot segmentation for these high-affinity DNA spots was then replaced with their identified top-20%-intensity regions in *maskTemp*. Weak-affinity spots inherited these 20% intensity regions from their nearest neighboring high-affinity spots. Together with the replaced spot segmentation for high-affinity spots, we have a final binary mask for DNA spot segmentation addressing the heterogeneous binding pattern, *maskFinal*.

The final spot fluorescence intensity time-trace for each DNA spot was computed as the difference between its mean intensity value from the top 20% of the pixels in the *maskFinal* segmented region and their corresponding local background mapped by *bkgMask*. These refined segmentation masks for DNA spots and local background (*maskFinal* and *bkgMask*) were uniformly applied to all image frames in one PBM experiment with a certain LacI variant; all frames were first subtracted with an offset frame captured before the introduction of LacI into the flow cell for the purpose of minimizing background noise. Consequently, the time-resolved fluorescence intensity trace for each valid DNA spot across association and dissociation phases in a PBM experiment involving one LacI variant was then obtained.

#### Post-processing and kinetic analysis of acquired PBM fluorescence time traces

Obtained DNA spot fluorescence time traces were first filtered and then grouped by corresponding DNA sequence. Spots were filtered according to four criteria: (i) Each spot’s initial local background must not exceed 400 fluorescence units (the background level during the association phase). This threshold ensures the exclusion of DNA spots that cannot be identified for their exact time points for the beginning of association. (ii) The maximum value, *f*_max_, of moving means (10 time point window size) of the spot intensity time trace needs to be > 0. (iii) The sum of the absolute differences between subsequent intensity values in the time-trace needs to be below a threshold, 85, because large frame-to-frame variability in the time trace is not consistent with LacI binding to DNA. (iv) Each DNA spot’s local background time trace was normalized to its *f*_max_, and only those normalized traces with fewer than six values falling outside of the range of −0.5–1.5 were kept. The spots that passed all four filters above were assigned to their corresponding unique DNA sequence group. DNA sequences with fewer than three valid spots from this initial filtering step were discarded from further analysis.

Next, the exact time points marking the beginning of association and dissociation were determined for each valid DNA spot. When the reaction buffer containing fluorescently labeled LacI protein flowed into the PBM chamber, the background fluorescence increased rapidly due to the presence of free protein in the imaging buffer. Conversely, when the LacI-containing buffer was replaced by the imaging buffer without protein, the background fluorescence rapidly decreased. To identify precisely when these buffer exchanges occurred for each DNA spot, we first obtained rough estimates using the average overall background intensity across all DNA spots on the whole array. Specifically, the time point where the largest increase happens in the overall background intensity time trace indicates the approximate universal start of the association phase, and the time point with the largest decrease indicates the universal start of the dissociation phase for all DNA spots. Then, for each individual DNA spot on this array, we refined these rough start time estimates. The average high-local-background fluorescence during the association phase of each DNA spot was computed by averaging the intensities from 10 consecutive time points (8.5 min), beginning 10 time points after the estimated universal start of association time point in its local background time-trace. Similarly, the low-local-background intensity during its dissociation phase was calculated. Each DNA spot’s local background fluorescence time trace, after being subtracted with the low-local-background value, was then normalized to its absolute difference between its own high- and low-local-background values. Then, the exact start time point index of association for each DNA spot was defined as its first time point index, *startIdx*, where the normalized local background fluorescence value exceeded 0.5. Similarly, the dissociation start was determined as the last time point index, *stopIdx*, meeting this criterion. After identifying the start and stop indices, DNA spots with unrealistic indices were excluded from analysis based on the following three criteria: (i) association to dissociation duration fewer than 50 time points recorded, (ii) *stopIdx* is beyond the selected range of corresponding time-frame with < *bkgLow* intensity, (iii) *startIdx *= 1, because this means the association on this DNA spot already started before we could capture it. For valid DNA spots that pass the above criteria, the precise association and dissociation times (*startTime* and *stopTime*) were then interpolated between their corresponding time points for frame index: *startIdx*-1 and *startIdx, stopIdx*-1, and *stopIdx*, respectively.

The binding and unbinding kinetics for each DNA spot were modeled by fitting their fluorescence time traces to exponential models. In the association phase, a single exponential curve is applied to fit the intensity data over a 30-min time window from the interpolated *startTime*. The association fit function was defined as.


\begin{eqnarray*}
f\left( t \right) = \left\{ {\begin{array}{@{}*{1}{c}@{}} {c,\begin{array}{@{}*{1}{c}@{}} {}\\ {} \end{array}\ t < 0}\\ {\frac{g}{b}\left( {1\ - \ {{e}^{ - bt}}} \right) + c,\ t \ge 0} \end{array}} \right.,
\end{eqnarray*}

where *g = k_a_* × [protein] × [DNA_tot_] × proteinFluor, where [protein] means the free concentration of LacI during the flow experiments, [DNA_tot_] means the total concentration of double stranded DNA on a DNA spot and proteinFluor means the mean fluorescence each protein gave, *b* = *k_a_* × [protein] + *k_d_*, and c is a constant. For the dissociation phase, a 15-min window after the interpolated *stopTime* was used. An inferred equilibrium fluorescence, *C*_max_, was calculated as ${{C}_{max}} = \frac{g}{b} + c$. During the first 15 min of dissociation, the DNA spot intensity curves are well-described by the sum of an exponential decrease and a constant. To estimate the dissociation rate constant, *k_d_*, we use the initial slope of the fitted curves. More specifically, two dissociation fitting models were employed for weak and strong affinity DNA spots, respectively. For weak-affinity DNA spots (those with computed maximum value out of their 10-frame moving means from their time trace <150 fluorescence units), 50 min prior to the dissociation start time were also added to the fitting window for better capturing the spot intensity at the dissociation start point. In this first scenario, the expanded fitting window used the function:


\begin{eqnarray*}
{\mathrm{\ }}f\left( t \right) = \left\{ {\begin{array}{@{}*{1}{c}@{}} {at + c,\begin{array}{@{}*{1}{c}@{}} {}\\ {} \end{array}\ t < 0}\\ {\frac{{g \cdot c}}{b}{{e}^{ - bt}} + c - \frac{{g \cdot c}}{b},\ t \ge 0} \end{array}} \right..
\end{eqnarray*}

Here, *c* is the spot intensity at the start of the dissociation. The initial slope of the dissociation curve, $f^{\prime}( 0 )\ = \ - g \cdot c$, and thus *g = k_d_*. For spots with higher intensities (≥150 fluorescence units), the constant *c* is fitted directly from the dissociation curve, and thus a simpler model was used:$f( t ) = \frac{{g \cdot c}}{b}{{e}^{ - bt}} + c( {1 - \frac{g}{b}} )$, where *g = k_d_*. The underlying reason for the constant offset is unknown.

To assess the reliability of the fitted *g* parameters from both the association and dissociation stages for a DNA spot intensity time trace, we calculated the CV by dividing the half width of the 67% confidence interval of the *g* parameter (approximately one standard deviation) by the corresponding g value for both fits. DNA spots for which the CV > 1 for either the association or the dissociation stage—indicating that the relative uncertainty was greater than the fitted parameter itself—were excluded from further analysis.

For each unique DNA sequence represented by at least three valid DNA spots in a LacI variant-PBM experiment, its averaged ‘equilibrium’ fluorescence (*f*_eq_) and corresponding SEM were computed based on *f*_max_ values from all its valid DNA spots. Finally, averaged kinetic parameters and their corresponding SEM were determined from fitted parameters as listed below for each valid DNA spot: the association parameter *g*, equal to *k_a_* × [protein] × [DNA tot] × proteinFluor; the inferred equilibrium fluorescence (*C*_max_); and the dissociation rate constant *k_d_*.

For data presented in Fig. 2 and Supplementary Fig. S1, the kinetics parameters for each LacI variant with a unique DNA sequence, its association-related parameter g = k_a_*C, C = [protein] × [DNA_tot_] × proteinFluor collects global experimental factors. All experiments were run with a fixed concentration of fluorescently labeled protein. In each replicate with two PBMs, the PBMs with printed ssDNA were synthesized into dsDNA in the same reaction, thus the [DNA_tot_] of all DNA spots on PBMs were assumed same within each replicate (this assumption is validated because no consistent spot-wise correlation were shown in one array for DNA spots of *O*_sym_, *O_1_* or *O_2_*, data not shown). Taken together, *C* cancels when we normalize k_a_*C with Wt-*k_a_*(*O*_sym_)*C within a side-by-side PBM experimental replicate. These within replicate relative *k_a_* values were then averaged across replicates to obtain the reported relative *k_a_* values.

#### Calculation for microscopic rates

As shown in Marklund *et al.* [31], the microscopic dissociation rate (*k*_off,µ_) and the maximum association rate constant (*k*_on,max_) can be inferred by applying the equation, *k_a_*= *k*_on,max_ - *k_d_* × *k*_on,max_/*k*_off,µ_. We fitted the above equation to the observed *k_a_, k_d_* relations shown in Supplementary Fig. S1A. The *k*_on,max_ ​value and its standard error (SE) were computed through the linear regression on *O_sym_​* single mutant sequences showing the top 35% *k_a_* among the sequences that have their *k_a_* and *k_d_* both satisfy their coefficient variation (CV) < 0.3. For estimating *k*_off,μ_, the above determined *k*_on,max_ was used in the equation: *k_a_*= *k*_on,max_ − *k_d_* × *k*_on,max_/*k*_off,µ_, which was applied to all valid *O*_sym_ single mutant sequences that have both *k_a_* and *k_d_* passed the CV < 0.3 filter. The mean and the standard error of the mean (SEM) of *k*_off,μ_ were then calculated after trimming the top and bottom 10% of the individual calculated k_off,μ_ values to remove the outlier values.

#### Strain construction for *in vivo* experiments

**For microscopy (single-molecule experiment):** The background strain with the wild type LacI, EL3498, was constructed by removing phi80 and its attachment site from JE107 [6] (*E. coli* BW25993 *lacI-mVenus* Δ(*p*_lac_*-lacZ*)::term)(see Supplementary Table S1 for a complete list of strains). Using DIRex [34], point mutations were introduced in the *lacI* gene of EL3498 to obtain strains, EL3518 with LacI(V52A), and EL3520 with LacI(Q55N) amino acid substitutions. Polymerase chain reaction (PCR) was performed using primer pairs, aslA-hom-cat-*O_sym_*-Fwd and aslA-hom-cat-*O_sym_*-Rev (see Supplementary Table S2) to obtain PCR product, lac*O_sym_* (sequences included in the reverse primer) along with Chloramphenicol cassette, herein written as, cat-lac*O_sym_*. As a template for Chloramphenicol resistance cassette, chromosomal DNA of strain EL8 was used. This PCR product was DpnI digested and further introduced into strain EL3498, using lambda red transformation [35] to obtain a strain with cat-*O*_sym_ in the *aslA-glmZ* intergenic region. The resulting strain was named EL4053. P1 transduction [36] was performed from strain EL4053 to transduce cat-lac*O_sym_* into the recipient strains, EL3498, EL3518, and EL3520 to obtain strains, EL4089, EL4242, and EL4243, respectively.

As we aimed to have only a few LacI molecules per cell in order to keep the fluorescence from nonbound LacI low, we reduced the expression of *lacI* in the above strains by changing the wild-type *p_lacI_* promoter to the *p*FAB138 from the Mutalik *et al.* promoter library [37]. A ribosome binding site [RBS from *p_lacI_* (wt)], was added after the promoter. A kanamycin resistance cassette was introduced upstream of the promoter sequence, transcribing in the opposite direction of the pFAB138 promoter. PCR primers were designed such that the primer, Comp1_Kan-Rv, has 40 bp homology at the *mhpA-mhpR* region followed by bases to amplify the kanamycin cassette from the reverse side (3′ end), while the other primer pair, Comp2_lacI 14 bp_RBS_p138-Fw contains bases to amplify the kanamycin resistance cassette from the forward side (5′ region), *p*FAB138, RBS and the first 14 bases of *lacI*. As a template for the kanamycin resistance cassette, chromosomal DNA of strain EL2620 was used. The obtained PCR product was DpnI-digested and introduced into EL3498, EL3518, and EL3520 by lambda-red transformation to yield the strains EL4210, EL4264, and EL4265, respectively. Thereafter, P1 transduction from EL4210 (donor) into EL3498 and EL4089 yielded the strains, EL4219 and EL4226, with and without cat-lac*O*_sym_, respectively. Similarly, P1 transduction from EL4264 (donor) into EL3518 and EL4242 yielded the strains EL4266 and EL4268, respectively. Furthermore, P1 transduction from strain EL4265 (donor) into EL3520 and EL4243, yielded the strains EL4267 and EL4269, respectively.

**For Miller assay:** Strains with LacI-mVenus variants were constructed based on the microscopy strains as described above. Using infusion cloning (Takara Bio), cat-*p*_lac_-lac*O*_sym_-*lacZ* was cloned on a R6K plasmid. Genomic DNA of EL409 was used as the source for *p*_lac_-lac*O*_sym_-*lacZ*. The resulting plasmid, pBS19, was sequence verified using primer pairs T7 Term_fwd and CAT-R. The insert of interest, herein written as cat-lac*O*_sym_-*lacZ*, contains the Chloramphenicol cassette upstream to the lac promoter sequence transcribing in the opposite direction, followed by lac*O*_sym_ and the entire *lacZ* gene. PCR was performed using plasmid template pBS19 and the primer pairs aslA-hom-cat-*O*_sym_-F and cat-*O_sym_*-lacZ_aslA_R. The PCR product was DpnI-digested and introduced at the *aslA-glmZ* intergenic region of strain EL4219, EL4266, and EL4267 using lambda red transformation [35] to obtain the strains, EL449, EL4493, and EL4495, respectively. To obtain a *lacI* knock-out mutant as a background strain for the Miller assays, we first exchanged *lacI* in the strain EL4219 with a selectable/counterselectable marker, *Acatsac1* [34]. Further, *Acatsac1* was replaced by a curing oligo tagatttaacgtataagagagtcaattcagggtggtgaatatggtgagcaagggcgaggagctgttcaccggggtggtgc, resulting in the strain EL4536. The PCR product, cat-lac*O_sym_*-*lacZ* from above, was further introduced at the *aslA-glmZ* intergenic region of EL4536 using lambda red transformation [35], resulting in the strain EL4580.

Strains for LacI-Halo variants, as used in DNA microarray experiments, were constructed similarly to above. We first exchanged the entire *mhpR-lacI-venus* in the strain EL3498 with the selectable/counterselectable marker *Acatsac1* [34]. The resulting intermediate strain was named EL4621. Alongside, using infusion cloning (Takara Bio), Kan^R^-*p*FAB138-*lacI-halo* or Kan^R^-*p*FAB138-*lacI*(V52A)*-halo* or Kan^R^-*p*FAB138-*lacI*(Q55N)*-halo* was cloned on R6K plasmid, resulting in the plasmids pBS20 (Kan^R^-*p*FAB138-*lacI-halo*), pBS21 (Kan^R^-*p*FAB138-*lacI*(V52A)*-halo)* or pBS22 (Kan^R^-*p*FAB138-*lacI*(Q55N)*-halo*) respectively. All three plasmids were sequence verified using primer pairs Seq_Halo_F, Seq_Halo_F and lacI mid rev3252. PCR was performed using plasmid templates, pBS20, pBS21, or pBS22, and primer pairs Comp–Kan–R and LacI_Halo_R. PCR products were digested with DpnI and introduced using lambda red transformation [35] into the intermediate strain EL4621 and selected on sucrose plates. The resulting sucrose-resistant recombinants were named EL4630, EL4632, and EL4634. Finally, the PCR product, cat-lac*O*_sym_-*lacZ* from above, was transformed using lambda red transformation [35] into the strains, EL4630, EL4632, and EL4634, resulting in the strains, EL4639, EL4641, and EL4643, respectively.

All above strains used in single molecule experiment and Miller assay were sequence verified; however, EL4643 showed a point mutation, 98C > 98T (Pro34 > Leu34), in the Halo-tag. We argue that this point mutation in halotag most likely does not influence the functionality of LacI in the Miller assay. Growth rate analyses were performed on the strains prepared for the Miller assays, all of which demonstrated comparable doubling times.

### Single-molecule tracking in living cells with microfluidics

#### Cell culture and preparation

Overnight cultures were grown in LB media at 37°C, diluted 1:500 in M9-glucose media (0.4% glucose, 0.4% RPMI 1640 Amino Acids Solution (Sigma–Aldrich, R7131), and 80 μg/ml Pluronic F-109 (Sigma–Aldrich, 542342), and incubated for 2–3 h at 30°C before loading into the microfluidic chip.

#### Microfluidic chip and Media flow control

A Polydimethylsiloxane microfluidic chip with cell trap-dimensions of 48 µm × 60 µm × 1 µm was used. Two different strains were loaded into opposite sides of the same microfluidic chip. In total 100 traps for each strain were imaged in each experiment. The microfluidic chip allowed for on-chip switching [38] between growth medium lacking Isopropyl β-D-1-thiogalactopyranoside (IPTG) to growth medium containing 0.3mM IPTG. Pressure in the chip was controlled using an in-house pressure regulator. Before the start of each association experiment, cells were exposed to 0.3 mM IPTG by switching pressures on the two media ports (from 90 to 240 mbar on the IPTG-containing media port and from 240 to 90 mbar on the IPTG-lacking media port) for at least 2 min.

#### Microscopy and imaging

Wide-field microscopy was performed with the following setup:

- Microscope: Nikon Ti2-E with 100×/1.45 NA oil immersion lens (CFI Plan APO lambda, Nikon).
- Illumination: Spectra III (Lumencor) for epi-fluorescence.
- Optical components: Dichroic mirror [ZT514rdc-UF2 (Chroma)], excitation filter [FF01-514/3(Chroma)], emission filter [ET550/50M (Semrock)].
- Camera: Kinetix (Teledyne Photometrics).
- Software: micro-Manager [39].

Fluorescence images (4-s exposure) and phase contrast images (80 ms exposure) were recorded. When capturing images, we alternated between the two strains with ∼5 s between images, and the cells on one side of the chip were captured every 15 s. Each experiment included multiple runs, imaging 20–25 traps per side. To minimize artefacts, we started the experiments on different sides of the chip. We began image acquisition 10 s before removing IPTG (switching pressures to 240 mbar for M9 media and 90 mbar for IPTG), ensuring synchronized capture of equilibrium binding (following 2-min IPTG exposure) and re-binding events post-IPTG washout. Experiments were performed at 30°C.

### Analysis of single-molecule experiments

#### Image analysis

Cells were segmented based on the phase contrast images using a U-Net convolutional neural network [40]. Fluorescent dots (mVenus molecules) were detected using a wavelet-based algorithm [41]: thresholding nonsignificant wavelet coefficients in the second á Trous wavelet plane. The threshold was defined as 5σ, where σ is the MAD estimate of the s.d. in the second wavelet plane [42]. The average number of dots per cell versus acquisition time (beginning with an initial measurement taken under steady-state (SS) with IPTG present, followed by subsequent time points recorded after IPTG removal through media switch) was collected to obtain binding curves. The average number of dots detected in the strains that did not contain the *O_sym_* operator was first subtracted from the average number of dots detected in the strains with the *O*_sym_ operator to avoid counting nonoperator binding into specific operator binding. Because images from the two strains were recorded with a 5-s offset, interpolation was applied to align the data before subtraction.

#### Kinetic analysis of LacI binding to O_sym_ after IPTG removal

To account for the sigmoidal shape of the binding curve, which is especially prominent in the Q55N LacI variant, we introduce a model in which IPTG efflux is explicitly modeled and IPTG binding to LacI inhibits it from switching into the specifically bound state. The model extends the three-state model from Marklund *et al.* [31], in which a LacI dimer can bind to the stretch of DNA that contains the *O_sym_* operator sequence. LacI is assumed to form a strong and stable dimer and this dimerization is not included in the dynamic model. Reference to LacI in the model description below implies the LacI dimer. While on the DNA, LacI can slide and possibly bind specifically to the *O*_sym_, or dissociate from DNA. The extension to the Marklund *et al.* model lies in that LacI also has the possibility of binding IPTG. IPTG is assumed to be able to bind LacI while it is free or is nonspecifically bound, but not when it is specifically bound to *O_sym_*. Limiting the number of DNA-bound states of LacI to only two, gives a manageable model with a manageable number of fitted parameters, but may not capture all aspects of the binding dynamics. The state where LacI is on top of the *O*_sym_ site, but not in its specifically bound conformation, a state to which IPTG can bind, is an example of a state which is not included in the model. A consequence of excluding this state is that the IPTG-induced dissociation from the specifically bound LacI becomes unrealistically slow. However, we deemed this limitation to have a small effect in our experiments where IPTG is excluded from the growth medium. In our model, IPTG binding is assumed not to change the properties of LacI nonspecifically bound to DNA, and the nonspecific binding to DNA is assumed not to change the IPTG affinity to LacI [43]. IPTG binding and release are assumed to be rapid as compared to LacI-DNA binding, such that the IPTG binding reaction can be treated as at equilibrium. Taken together, the LacI binding can be described by the reaction scheme below, same as in Fig. 4B:


\begin{eqnarray*}
\begin{array}{@{}*{5}{c}@{}} {O + LI}&{\underset{{{{k}_{off,M}}}}{\overset{{{{k}_{on,\max }}}}{\rightleftharpoons}}}&{OLI}&\,&\,\\ { \uparrow \downarrow {{K}_i}}&\,&{ \uparrow \downarrow {{K}_i}}&\,&\,\\ {O + L + I}&{\underset{{{{k}_{off,M}}}}{\overset{{{{k}_{on,\max }}}}{\rightleftharpoons}}}&{OL + I}&{\underset{{{{k}_{off,\mu }}}}{\overset{{{{k}_{on,\mu }}}}{\rightleftharpoons}}}&{O{{L}_S}} \end{array}
\end{eqnarray*}

where:

- **O**: the specific operator site (*O_sym_*) in the genome.
- **L**: LacI protein
- **OL**: the complex of LacI bound to the operator nonspecifically (corresponding to the testing state in the 3-states model stated in [31].
- **I**: IPTG
- **OL_S_**: the complex of LacI bound to the operator specifically
- *k_on,max_*: the on rate into testing state from unbound state to operator site
- *k_off,M_*: dissociation rate from the testing (nonspecifically bound to the operator site) state to the state of unbound with operator site
- *K_i_*: IPTG binding constant
- *k_on,μ_*: the on rate of testing state LacI transition into the specific-bound state on the operator.
- *k_off,μ_*: the off rate of specific-bound state LacI go back into the testing state.

The decrease in the intracellular IPTG concentration is modelled using first-order kinetics, and, as in Marklund *et al.*, the nonspecifically bound *OL*-state is assumed to be very sparsely populated, such that the specifically bound state and the IPTG concentration can be described by the following set of ODEs:


\begin{eqnarray*}
{\left\{ {\begin{array}{@{}*{1}{c}@{}} {\frac{{d\left[ {O{{L}_S}} \right]}}{{dt}}\ = {{{\tilde{k}}}_{on,max}}\ {{p}_{tot}}\left( {\left[ I \right]} \right)\left( {{{O}_{tot}} - \left[ {O{{L}_S}} \right]} \right) - {{k}_{off,\mu }}\left( {1 - {{p}_{tot}}\left( {\left[ I \right]} \right)} \right)\left[ {O{{L}_S}} \right]}\\ {\frac{{d\left[ I \right]}}{{dt}}\ = \ - \gamma \left[ I \right]\ } \end{array}} \right.}
\end{eqnarray*}

Where O_tot_ is the total concentration of O_sym_ operator in the cell, ${{\tilde{k}}_{on,max}} = {{k}_{on,max}}[ L ]$ and the IPTG-concentration([I])-dependent probability of forming the $O{{L}_S}$ complex when in the $OL$ state, ${{p}_{tot}}( {[ I ]} )$ is.


\begin{eqnarray*}
{{p}_{tot}}\left( {\left[ I \right]} \right) = \frac{{{{k}_{on,\mu }} \cdot \frac{{{{K}_i}}}{{{{K}_i} + \left[ I \right]}}}}{{{{k}_{off,M}} + {{k}_{on,\mu }} \cdot \frac{{{{K}_i}}}{{{{K}_i} + \left[ I \right]}}}} = \frac{1}{{1 + \left( {\frac{1}{{{{p}_{tot}}\left( {\left[ I \right] = 0} \right)}} - 1} \right) \cdot \frac{{{{K}_i} + \left[ I \right]}}{{{{K}_i}}}}}
\end{eqnarray*}

The free LacI concentration is assumed to be approximately constant throughout the experiment.

In the regression, the following parameters are fitted: ${{p}_{tot}}( {[ I ] = 0} )\ = \ \frac{{{{k}_{on,\mu }}}}{{{{k}_{on,\mu }} + {{k}_{off,M}}}}$,${{k}_a} = \ \ {{\tilde{k}}_{on,max}} \cdot {{p}_{tot}}( {[ I ] = 0} )$, ${{\tilde{K}}_i} = {{K}_i}/{{i}_0}$, O_tot_, and γ. Here ${{i}_0}$ is the initial IPTG concentration before the switch to media without IPTG. In the regression, the relation between association and dissociation rates is locked to the Miller measurements in the same strain using the relation


\begin{eqnarray*}
\phi - 1 = \frac{{{{{\tilde{k}}}_{on,max}}{{k}_{on,\ \mu }}\ }}{{{{k}_{off,M}}{{k}_{off,\ \mu }}}}
\end{eqnarray*}

Note that the fitted *in vivo* association rate, k_a_, encompasses macromolecular crowding, 3D diffusion, 1D sliding/hopping, nonoperator binding, and re-association; i.e. it reflects the net rate of forming a detectable specific complex at O_sym_  *in vivo*.

#### Miller assay

Overnight cultures were grown in M9-glucose medium with and without 0.3 mM IPTG at 37°C. The overnight cultures were diluted (1:300) in fresh medium and grown to an OD_450_ of 0.5–0.6, where the exact value was recorded for each sample. For each combination of conditions (i.e. ±IPTG) and LacI variant (i.e. Wt, Q55N, and V52A), five replicates of 100 µl cell culture samples were prepared. Each culture sample was lysed in 700 µl Z buffer (60 mM Na_2_HPO_4_, 40 mM NaH_2_PO_4_, 10 mM KCl, 1 mM MgSO_4_, pH 7, with freshly added 10 mM dithiothreitol and 0.1% sodium dodecyl sulphate) and 150 µl chloroform. The mixture was then vortexed for at least 5 s and incubated at 28°C for 5 min to ensure complete lysis. The enzymatic reaction of β-galactosidase was initiated by adding 100 µl of ortho-Nitrophenyl-β-galactoside (4 mg/ml) substrate solution, allowing the yellow color (from the product o-nitrophenol) to develop. The reaction was terminated by the addition of 400 μl 1 M Na₂CO₃ at five different reaction stop times for each condition of a given LacI variant. In the presence of IPTG in the growth medium, the reaction times ranged from 5–63 min for the DelLacI strain, 13–72 min for the Wt-LacI strain, 10–69 min for the Q55N-LacI strain, and 21–143 min for the V52A-LacI strain. Without IPTG, the reaction times were longer, ranging from 6–65 min for DelLacI, 30–150 min for Wt-LacI, 12–71 min for Q55N-LacI, and 53–304 min for V52A-LacI. Shortly after each reaction was terminated, the mixtures were transferred to microfuge tubes, centrifuged at 12 000 × *g* for 1 min, and the supernatants were collected for Abs₄₂₀ measurements.

Miller Units were calculated by determining the slope of Abs₄₂₀ values over reaction time using linear regression, normalized for culture density and sample volume. The formula used was: Miller Units = [1000 × SLOPE(Abs₄₂₀, Reaction Time)]/(Abs_450_ × 0.1), where the slope of Abs₄₂₀ values (reflecting β-galactosidase activity) versus reaction time was scaled by a factor of 1000 and divided by the absorbance at 450 nm (Abs_450_) to account for cell density, as well as the sample cell culture volume of 0.1 ml.

#### Representing repression strength and O_sym_ occupancy via Miller Units

To isolate the repressive effect of LacI-venus variants, Miller Units for LacI-venus variants were normalized against the ΔLacI-venus strain (lacking LacI, hence completely nonrepressed β-gal production). At steady state, the [β-gal] level depends on the operator’s occupancy: in LacI variants, [β-gal] = *γ*⋅*P*_free_/μ (where *γ* = maximal production rate, μ = degradation rate of β-gal), while in ΔLacI (where the operator is always free, *P*_free_ = 1), [β-gal] = *γ*/μ. Normalizing Miller Units (which is proportional to [β-gal] in cells) gives:$\frac{{{\mathrm{ Miller}}\ {\mathrm{ Units}}\ ( {{\mathrm{ LacI}} - {\mathrm{ venus}}\ {\mathrm{ variant}}} )}}{{{\mathrm{ Miller}}\ {\mathrm{ Units}}\ ( {{\mathrm{ DelLacI}}} )}}\ = \frac{{\gamma \cdot {{P}_{{free}}}/\mu }}{{\gamma /\mu }}\ = \ {{P}_{{\mathrm{ free}}}},$ directly reflecting the probability of the lac operator (*O*_sym_) being unoccupied, *P*_free_ = 1 − *P*_bound_ = normalized Miller Units. Then the inverse of normalized Miller Units (*ϕ*), *ϕ* = 1/(1 − *P*_bound_), shows the correlation with repression strength: higher *P*_bound_ leads to higher *ϕ. O*_sym_ occupancy can thus be quantified as *P*_bound_ = $1 - \ \frac{1}{\phi }$.

#### Electricity mobility shift assay

Electrophoretic mobility shift assays (EMSAs) were performed to analyze the difference in the affinities of *O*_sym_ DNA with LacI-Halo variants (the Wt and Q55N mutant, same purified and protein labeled as in the PBM experiment stated above) in different salt concentrations. Then, 1 nM 5′IRdye700-labeled *O*_sym_ containing 60 bp DNA (same nt sequence as in PBM) were used in binding reactions with varying concentrations of LacI proteins (ranging from 0 to 500 nM) at different NaCl concentrations (20, 100, and 200 mM). Binding reactions were carried out in a 12 µl volume containing 1 nM DNA, 1× imaging buffer (same buffer as stated in the PBM experiment, except varying NaCl concentration). Negative controls included reactions without DNA or reactions without protein. The reaction mixtures were incubated at room temperature for 1 h. Samples were loaded onto 5% Mini-PROTEAN^®^ Tris/Boric acid/EDTA (TBE) gels (15 wells)(Bio-Rad). The gels and running buffer (0.5× TBE) were pre-chilled and run at 200 V for 35 min to separate the protein–DNA complexes from free DNA. The gels were then imaged with Chemidoc (Bio-Rad) and analyzed with Imagelab for quantifying the LacI-bound fraction of DNA.

## Results

### *In silico* evaluation of hinge helix propensity in LacI variants

In the LacI dimer the hinge regions connect the DBDs and the effector (allo-lactose/IPTG)-binding core domains. They are helices in the LacI crystal structure when bound to operator DNA (PDB ID: 1EFA) [44]. Additionally, a NMR study of a truncated construct containing only the DBDs and hinges shows that the hinge regions are disordered in the free state and when bound to nonoperator DNA. They only become helical when bound to operator DNA [27, 45]. The structural plasticity in the hinge regions supports the hypothesis that the switching between the search and the recognition conformations is associated with the folding of the hinge helix (Fig. 1A) [46, 47]. Guided by this hypothesis we selected five single amino acid substitutions that were expected [29] (Fig. 1C) to change the propensity of forming a helix. In the selection of mutants we tried to minimize unwanted side-effects in terms of protein structure and DNA binding by choosing amino acids with similar physio-chemical properties as contained in the wild-type. To characterize the hinge helix propensity in the different LacI variants we first studied the hinge helix region as a short peptide in solution. Here predictions of helical propensities for peptide amino-acid sequences containing the hinge helix and its flanking sequences (18 amino-acids in total, N46-L63) were generated using the empirically parameterized Agadir software [29, 30] for each LacI variant (Fig. 1D). As expected from intrinsic helical tendencies (Fig. 1C) [29], which are part of the Agadir model, the software predicted that the mutations Q55N, Q54N, and L56V decrease whereas G58A increases helix propensity compared to wt. The expected increase in helix propensity of the Valine to Alanine mutation (Fig. 1C) should primarily be located in the Arg51-Glu54 part of the helix. Secondly, the hinge helix propensity of the wt, as well as the Q55N, G58A, and V52A variants were investigated using all-atom MD simulations of the LacI dimer. The Q54N and L56V variants were not selected for MD simulation due to the similarity to the Q55N variant according to the Agadir helicity predictions. Quantifying the transition between search and recognition states using all atom MD simulations of LacI bound to operator DNA is computationally intractable [48] and we have previously shown [21] that enhanced sampling of the transition is nontrivial. Here we, instead, simulated the free LacI dimer in solution and quantified when the hinge regions form helices, defined as having an C-alpha RMS deviation from ideal helix of residues R51–A57 < 0.1 nm (see the ‘Materials and methods’ section for details). The wt simulations were started from the crystal structure with the PDB-ID 1EFA [15] from which DNA and ONPF were removed and the mutant simulations were started with the corresponding mutation introduced in the wt structure. All simulations were performed in five replicas. See the ‘Materials and methods’ section for details of the MD simulations. For all of the LacI variants, the fraction of hinges in helical form in the five replicate simulations decreases for about 300 ns until reaching a plateau (Fig. 1E). In line with previous MD simulations of V52A [26], the fraction of hinges in helical form decreased slower for the V52A variant as compared to Wt. At the plateau, the distribution of hinge helix fractions for the V52A mutant have a larger mean than the Wt distribution with a *P*-value of 0.0035 test using Welch’s test (see the ‘Materials and methods’ section). Here the distributions were made by sampling the time trajectories every 15 ns to reduce correlation between the samples (Supplementary Fig. S3B). The helical fraction of the G58A mutant also decreased slower than Wt and the plateau distribution has a larger mean than Wt with a *P*-value of 0.0007. The Q55N mutant, on the other hand, showed similar behaviour as wt and only a *P*-value of 0.07 for the mean Q55N distribution being lower than Wt. The reason for the negligible effect in the Q55N mutant in the MD simulation as compared to the helix predictions for the peptide alone is currently unknown. We do not expect the lack of an effect in the helicity of the Q55N hinge to be due to force field parameter bias in the MD simulations, since previous results using a similar forcefield [49] have shown good agreement between estimates of helicity in estimates of helicity in MD and experiments experiments for short peptides. Rather the expected decrease for Q55N (Figs. 2C and D) could be masked by the uncertainty estimate, based on the five replicates. Taken together these results from both the short peptide helix prediction (FIg. 2D) and the MD simulation of the full protein (Fig. 2E) indicate that Q55N has a bias towards the search conformation and the G58A and V52A are biased towards the recognition confirmation. Although G58A displayed increased helix propensity in simulations, the initial *in vitro* experimental screening revealed similar DNA binding ability of G58A and Q55N to operators (Supplementary Fig. S8). In relation to this Suckow, J *et al.*, 1996 [50] showed that substitutions at G58 predominantly yield an I⁻ phenotype, i.e. constitutive lac operon expression caused by a loss of functional repression *in vivo*, whereas substitutions at Gln55 lead to milder defects. This indicates that changes at Gly58 tend to severely compromise LacI function, while Gln55 variants can retain substantial activity. Based on these results, the Q55N and V52A were selected as examples of mutants having decreased or increased hinge helix propensities and thus, expected either a deceased or an increased bias towards the recognition conformation of LacI. The G58A mutant was not selected for further investigations, although its limited binding raises the question of whether helix formation is the most critical parameter guiding binding.

### High-throughput *in vitro* binding kinetics reveal specificity–stability trade-offs

To test how the selected mutations impact LacI’s binding strength to different DNA sequences and hence its specificity, we first measured the *in vitro* association and dissociation rates of selected variants with 2479 DNA sequences, including the natural *O_1_, O_2_* operators, the artificially strong *O_sym_* operator, and their single and double mutants using protein-binding microarrays (PBMs) [31, 51] (Fig. 2). We mounted two PBMs in adjacent flow cells on a microarray slide (Supplementary Fig. S6). Wt-LacI was flowed over one of the PBMs while a LacI mutant was flowed over the other. Both proteins were fluorescently labeled. The binding dynamics for each LacI protein were measured by introducing the reaction buffer with LacI molecules (association phase) and subsequently introducing the reaction buffer without LacI molecules (dissociation phase). This process allowed us to capture the time-dependent increase (Fig. 2A and B, association) and decrease (Fig. 2A and B, dissociation) in binding signals. Relative association rates (*k_a_*​) were obtained by fitting association curves and normalizing the fitted parameter to Wt-LacI binding to *O*_sym_​ in the same experiment. Dissociation rate constants (*k_d​_*) were extracted from separate fits of the dissociation curves (see details in the ‘Materials and methods’ section).

The V52A-LacI mutant associates faster than the Wt-LacI to most mutated operator sequences (as shown in Fig. 2A and C, and Supplementary Fig. S4A). The deviation between V52A and Wt, on average, increases for sequences with lower association rates. The dissociation rate for V52A is slower than that for Wt from all sequences (Fig. 2D and Supplementary Fig. S4B), with a similar fold-change between V52A and Wt for all sequences. As a consequence, V52A-LacI has lower specificity (Fig. 2F) and generally a lower equilibrium dissociation constant, *K_D_*, as compared to Wt-LacI (Fig. 2E and Supplementary Fig. S4C). The shift towards higher affinity to the most of DNA sequences in the V52A-LacI mutant is consistent with the hypothesis that increased helix propensity gives more stable binding.

The Q55N-LacI mutant, where the hinge region is expected to be less helical as compared to wt, shows a slower association rate as compared to Wt-LacI for most sequences, where the deviation from Wt-LacI, on average, increases with lower association rate. Q55N-LacI shows faster dissociation from most sequences compared to Wt-LacI, with a fold change similar for most sequences. The shifts in association and dissociation rate constants as compared to Wt for the Q55N mutant result in an increased specificity (Fig. 2F), but a reduced binding strength for most sequences, including *O*_sym_ (Fig. 2E). The slow association and rapid dissociation for the Q55N mutant cause many weak binding sequences to fall below our detection limit. The *in vitro* data reveal that Q55N-LacI has a higher affinity to one of the *O*_sym_ single base-pair mutants than to the *O*_sym_ operator itself (Fig. 2E). However, this *O*_sym_ single base-pair mutant is absent from the wt *E. coli* genome; the closest sequence in the wt *E. coli* genome differs by 3 base-pairs from *O*_sym_ (Supplementary Table S4). In conclusion, if *O*_sym_ were to be introduced into the wt *E. coli* genome, Q55N-LacI would still recognize it with the highest affinity.

The *in vitro* results agree with a conformation-switch model, in which the changes in preference for the search or recognition conformations impact the specificity and binding stability of LacI to *O*_sym_. Biasing the hinge region of LacI towards adopting the helical (recognition) conformation stabilises the binding on operator sequences, but it also elevates the binding probability on nonoperator sequences, thereby reducing DNA specificity (and vice versa). The remaining question is how the propensity of forming helices in the hinge region of LacI affects its target DNA search speed in the intercellular context with all competing nonoperator DNA sequences present in the genome. Based on extrapolation from the *in vitro* data, predicting the operator access time of Q55N-LacI in relation to that of Wt-LacI is straightforward. Q55N-LacI interacts poorly with any sequences more than 1 base pair mutation away from the *O*_s_*_ym_*. Since Q55N should have a lower probability adopting the recognition conformation on nonoperator sequences the operator access time should be short, possibly shorter than that of Wt-LacI. However, the prediction for the total *O*_sym_ occupancy *in vivo* is not as straightforward, because the *in vitro* dissociation rate of Q55N-LacI from the *O*_sym_ operator is faster than that of Wt-LacI, which, together with potentially shorter operator access time, makes it hard to predict. The specificity of V52A-LacI is lower than that of Wt-LacI for near-operator sequences. This means that *in vivo*, the operator access time may be impacted by competing binding to nonoperator sequences, i.e. the operator access time could be longer than that for Wt-LacI. However, dissociation from *O_sym_* is slower, again making the *in vivo* SS binding uncertain without live-cell measurements.

### *In vivo* measurements reveal weak coupling between specificity and search speed

**Figure 3.**
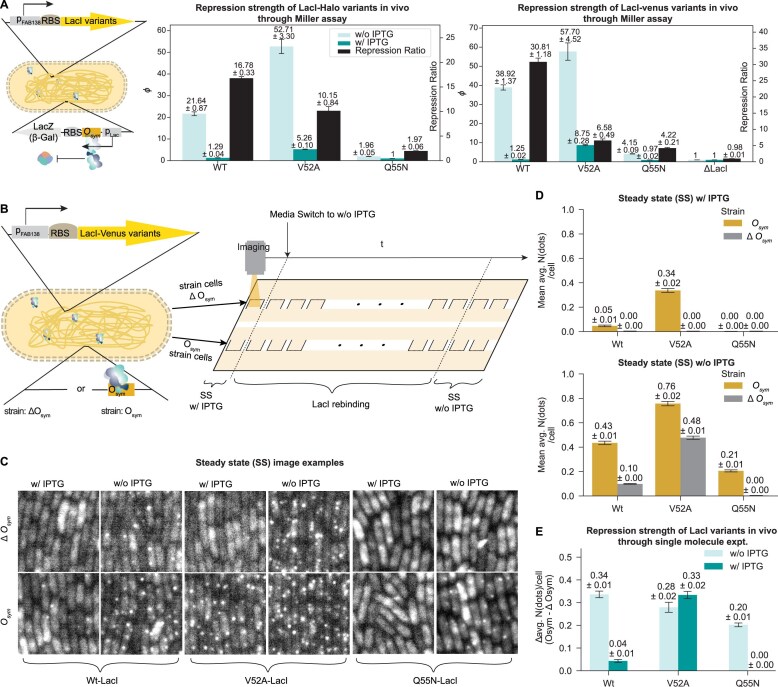
Steady state binding of LacI variants *in vivo* [(**A**) Miller assay, (**B–E**) Single-molecule experiments]. (**A**) Repression strength (1/Normalized Miller Units, denoted as *ϕ*) for LacI-halo variants (*middle*) and LacI-venus variants (*right*) through Miller assay. The β-gal activity (Miller Units) was measured under the control of the *O_sym_* operator with three LacI protein variants expressed from the *E. coli* genome (*left*). *ϕ* indicates the repression strength of each LacI variant on *O*_sym_. For the LacI-halo strains, as shown in the left bar plot, the Q55N variant with IPTG was used as a control, representing a state of no repression (where *ϕ* = 1). For the LacI-venus strains, as shown in the right bar plot, the ΔLacI variant served as the control for each state without (w/o) IPTG or With (w/) IPTG. All LacI variants were tested in three biological replicates. Data are presented as mean ± SEM. (**B**) A schematic overview of the single-molecule experimental workflow conducted on a microfluidic chip. Two isogenic *E. coli* strains, both expressing a certain Venus-tagged LacI variant but differing in their genomic presence or absence of the *O*_sym_ operator sequence, were independently introduced into fluidically connected cell chambers (labeled as squares open on one side), which are positioned on opposite sides of the chip. This setup allows strains to have simultaneous access to the shared growth media. Initially, chambers were exposed to media containing 0.3 mM IPTG. Fluorescence imaging was performed to capture the SS LacI binding in each strain under the IPTG-supplemented condition (SS w/ IPTG). Following this, media was exchanged to IPTG-free media, and time-lapse fluorescence imaging subsequently captured downstream chambers on both sides of the chip to quantify LacI rebinding kinetics until a new SS was established in media without IPTG (SS w/o IPTG). (**C**) Fluorescence images of cells expressing three LacI variants at steady state under two conditions: without IPTG (w/o IPTG) and with IPTG (w/ IPTG). Columns correspond to the IPTG condition, and rows correspond to two genetic backgrounds: one with the *O*_sym_ operator sequence integrated into the genome and one in which this operator is not present (Δ*O*_sym_). For each LacI variant, the paired images of the *O*_sym_ and Δ*O*_sym_ strains are indicated by brackets. (**D**) Detected average number of binding dots per cell for LacI variants in strains with or without *O*_sym_, in the steady states with IPTG (*top*) or without (*bottom*). (**E**) The *in vivo* repression strength of LacI variants to the *O*_sym_ operator is expressed as the average number of specifically bound LacI molecules, which is calculated by subtracting the average number of dots per cell in the Δ*O*_sym_ control strain from that in the *O*_sym_ containing strain measured at steady state, both with IPTG (w/ IPTG) and without IPTG (w/o IPTG).

To measure the *in vivo* SS binding of the LacI variants to *O*_sym_, we constructed strains in which the expression of β-galactosidase (β-gal) is regulated by LacI binding to *O*_sym_ (Fig. 3A). The intracellular β-gal concentration can then be measured using the Miller assay [52] and thus be used to estimate the SS LacI binding to *O*_sym_. We determined the *in vivo* SS binding to *O*_sym_ with the Miller assay for β-gal activity using the same halo-tagged dimer version of LacI as in the *in vitro* experiment. If the repressor is bound at the operator, the expression is off, which makes the activity proportional to the fraction of time the operator is unoccupied [52].

The Miller units are directly proportional to the intracellular concentration of β-gal. For the strains carrying the *O*_sym_ operator in the genome, the relationship between measured Miller units and the chance of the *O*_sym_ operator being bound by LacI can be written as: Miller units ∝ [β-gal] ∝ *P*_free_ (probability of *O*_sym_ being free). *P*_free_ can be calculated as *P*_free_ = $\frac{{{{t}_{{\mathrm{ free}}}}}}{{{{t}_{{\mathrm{ free}}}}\ + \ {{t}_{{\mathrm{ bound}}}}}}$ = $\frac{{1/\ {{k}_a}}}{{1/\ {{k}_a} + \ 1/{{k}_d}}}$ and the inverse of the normalized Miller units, abbreviated as *ϕ*, is then equal to $1 + \frac{{{{k}_a}}}{{{{k}_d}}}$. This implies that the ratios of *ϕ* correlate to the ratio of affinities (1/*K_D_*), such that $\frac{{\phi ( {Wt - \textit{LacI}} ) - 1}}{{\phi ( {Mut - \textit{LacI}} ) - 1}} = \frac{{1/{{K}_{D,Wt}}}}{{1/{{K}_{D,Mut}}}}$, assuming similar free intracellular LacI concentrations across the variants (controlled by the same promoter).

To estimate the *in vivo* association rate based on the SS binding estimate from the Miller assay, we need an estimate of the dissociation rate. If the relative dissociation rates of Wt-LacI and V52A-LacI observed *in vitro* (Fig. 2E) are similar to *in vivo*, the dissociation rate of V52A-LacI would be roughly 2.4 times slower than that of Wt-LacI. *In vivo*, V52A-LacI repressed the expression of β-gal ∼2.5, (52.71–1)/(21.64–1), times stronger than Wt-LacI (Fig. 3A, left bar plot). The close agreement between these values suggests that V52A-LacI may search equally fast as Wt-LacI *in vivo*. This would imply that V52A-LacI is not slowed down much by interactions with nonoperator DNA. This interpretation can, however, be questioned based on the microscopy experiments introduced below, which is described further in the discussion. Importantly, but independently of the search speed, V52A-LacI represses β-gal expression to a large extent also in the presence of IPTG, where the expression of β-gal is only 11% of that of the ∆LacI strain.

For the Q55N-LacI mutant, the binding strength to *O*_sym_ compared with Wt-LacI *in vivo* decreases by a factor of ∼20, while its *in vitro* dissociation rate only increases by a factor of ∼3. Assuming that this ∼3-fold ratio in dissociation rates of Wt-LacI and Q55N-LacI remain similar *in vivo*, this modest kinetic difference alone cannot account for the ∼20-fold difference in repression strength. One possible explanation would be an increased search time for Q55N-LacI *in vivo* compared to Wt-LacI. However, this interpretation is counterintuitive, as Q55N-LacI exhibits higher sequence specificity for *O_sym_* than Wt-LacI *in vitro*, which would be expected to facilitate rather than hinder target search. This highlights the need for a direct measurement of the search time *in vivo*.

To directly visualize the *in vivo* SS binding results, as assessed in the Miller assay above, and also to measure the *in vivo* O_sym_ operator search time, i.e. it takes for the LacI variants to access and specifically bind to the *O*_sym_ operator, we used the single-molecule method we previously described in Hammar *et al.* [6]. Following Hammar *et al.*, we expressed LacI as a translational fusion to the fluorescent protein Venus at only a few molecules per cell in a strain carrying a single *O*_sym_ in the genome (Fig. 3B). The operator is not regulating any promoter in this strain. The exact number of LacI-venus molecules in the cell is not critical as long as it is the same for the different variants, and the total fluorescence from freely diffusing molecules is sufficiently low to allow the observation of one chromosomally bound dimer as a diffraction-limited fluorescent spot when we use a long (4s) exposure time. The rationale is that only the specifically bound molecules give a signal over the background since the emission light from the nonbound molecules is blurred by diffusion. The bacteria are grown in a microfluidic device that allows for rapid media switches while imaging the two types of growing cells on the microscope (Fig. 3B) [53].

First, we quantified the SS *in vivo* binding of each LacI variant under two IPTG conditions—with IPTG (SS w/ IPTG) and long after IPTG removal (SS w/o IPTG)—in two genomic backgrounds: one with the *O*_sym_ operator (strain type: *O*_sym_) in the genome and one without (strain type: ∆*O*_sym_), as illustrated in Fig. 3B. Example images taken in SS are shown in Fig. 3C for different LacI variants. When spots are observed in strains without the specific operator sites, these correspond to long-lasting (>4s) binding to nonoperator sequences. Figure 3D shows the corresponding quantification of the average number of detected binding spots per cell, under SS conditions with or without IPTG. In SS w/ IPTG, Wt-LacI shows no spots in the ∆*O*_sym_ strain and a small number of spots due to specifically bound LacI in the *O*_sym_ strain. In SS w/o IPTG, there are some binding on non-*O*_sym_ DNA sequences in the ∆*O_sym_* strain, but the *O*_sym_ strain displays more binding events. V52A-LacI in SS w/o IPTG displays more binding to non-*O*_sym_ DNA sequences than the other two LacI variants. This is in line with the stronger binding of the V52A-LacI to operator mutants *in vitro* as compared to Wt-LacI. While IPTG reduces the V52A-LacI binding below the detection limit when there is no *O_sym_* in the genome, it is less effective at reducing the number of bound LacI molecules when *O*_sym_ is present in the genome (Fig. 3D). The large *O*_sym_ occupancy by V52A-LacI even in the presence of IPTG corroborates the Miller assay results, with which the *O*_sym_ occupancy can be calculated as *p*_bound_ = $1 - \ \frac{1}{\phi }$, see derivation in the ‘Materials and methods’ section. The calculation gives a probability of 89% (i.e. 1 − 1/8.75 ≈ 89%) that *O*_sym_ is bound by V52A-LacI in the presence of IPTG, this loss of inducibility phenotype for V52A mutant was also reported in previous studies [50, 54]. Q55N-LacI, on the other hand, only displays binding events when there is *O*_sym_ in the genome and is fully dissociated by IPTG. This is also expected from the *in vitro* experiments and the Miller assay data.

Next, we evaluated the dynamics of target search for Wt-LacI and Q55N-LacI just after removing IPTG (through media switch) in single-molecule experiments. The experiment could not be performed for V52A-LacI, because the *O*_sym_ occupancy by V52A-LacI remained high even with IPTG present, as seen in Fig. 3E. Any binding increase after IPTG removal likely comes from its binding to nonoperator sequences. The Miller assay also shows that *O*_sym_ occupancy in the V52A-LacI strain increases from 89% with IPTG to 98% (1–1/*ϕ* = 1 - 1/57.7 ∼ 98%) without IPTG. In contrast, for the *O*_sym_ operator, occupancy by Q55N-LacI is changing from close to zero to 59% of that of Wt-LacI, as shown in Fig. 3E (0.20/0.34 ≈ 59%). Therefore, by removing IPTG from the growth medium, we can directly measure the Q55N-LacI binding rate using time-lapse imaging, similar to our previous studies with Wt-LacI [3, 6].

**Figure 4.**
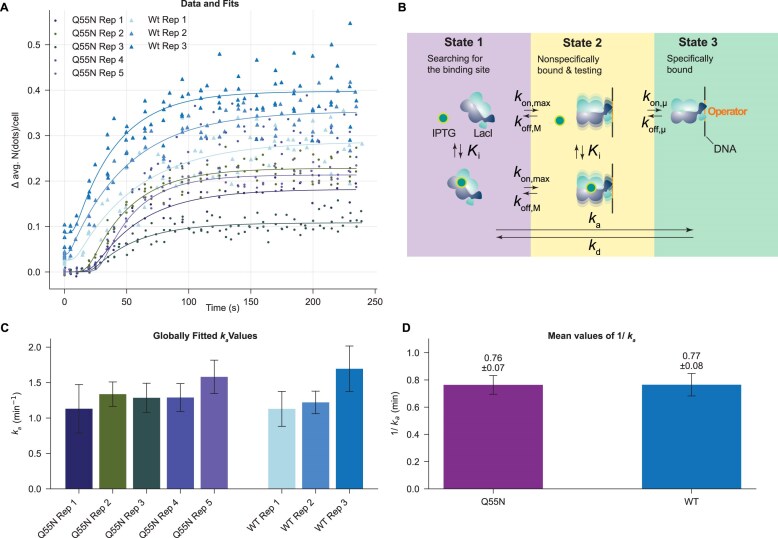
Binding kinetics of LacI variants to the *O*_sym_ operator *in vivo* over time after IPTG removal. (**A**) Binding is quantified by the difference in fluorescent spots per cell between *O*_sym_-containing strains (*O*_sym_) and control strains lacking *O*_sym_ (Δ*O*_sym_), in the same way as in Fig. 3E. The binding curves were fitted using the IPTG-integrated three-state model (solid lines; see model in panel (B) and details about fitting in the ‘Materials and methods’ section). (**B**) Three-state model, taken from Marklund *et al.* [31], integrated with IPTG. Microscopic rates entering and exiting from one state to another are labeled at the cross sections of states, *K_i_* is the equilibrium dissociation constant for the interaction between IPTG and the LacI dimer that is either in state 1 or state 2. Macroscopic association rate (*k_a_*) and dissociation rate (*k_d_*) are labeled out as for a LacI dimer from being far away from its target operator to being specifically bound to its operator. Parameterization and fitting are described in the ‘Materials and methods’ section. (**C**) Bar plots of fitted parameter: *k_a_* value with SE for each individual replicate for Wt-LacI and Q55N-LacI, respectively. (**D**) Average *in vivo* search times for Q55N–LacI and Wt-LacI dimers in a cell targeting a single *O*_sym_ operator in the bacterial genome. This time is calculated as the inverse of the averaged fitted *k_a_*, with error bars showing the propagated standard error.i

In Fig. 4A, binding of Wt-LacI and Q55N-LacI to the *O*_sym_ operator over time after IPTG removal is shown as the difference in the average number of detected spots per cell in strains with *O*_sym_ in the genome compared to the ∆*O_sym_* strain. To analyze the specific binding kinetics of the LacI variants to the genomic *O*_sym_ site after IPTG removal, we used a version of the kinetic model used in Marklund *et al.* [31], now extended to include IPTG. In this extended model (Fig. 4B), LacI is not allowed to form the specifically bound state if it is bound to IPTG (see more details in the ‘Materials and methods’ section). The change of intracellular IPTG concentration in the model continuously decreases due to the net IPTG efflux after switching to media lacking IPTG. LacI is controlled under the same promoter as in the Miller assay, thus the binding affinities measured using the Miller assay are assumed to be valid also for the *in vivo* kinetics measurement, because the ratio of *O*_sym_ occupancy between Q55N-LacI and Wt-LacI is measured to be in close agreement in the Miller assay and the single-molecule experiments. In the Miller assay, the *O*_sym_ occupancy for Q55N-LacI to Wt-LacI is (1–1/4.15)/(1–1/38.92) ≈ 0.78. In the single-molecule experiments, the ratio is calculated as above, 0.20/0.34 ≈ 0.59. With the two assumptions above, we fitted the Fig. 4B model to the binding data in Fig. 4A, allowing us to estimate the average *k_a_* to the *O*_sym_ operator *in vivo*. We find that the average time for a single *O*_sym_ operator in the bacterial genome to be found and bound by LacI is around 0.7 min in both the Wt-LacI and the Q55N-LacI containing strains (Fig. 4D). This indicates that making LacI prefer the search conformation does not accelerate its association rate to the operator site by minimizing nonoperator interactions. Instead, the primary effect of making LacI prefer the search conformation appears to impact much more on the dissociation rate with its target operator: since Q55N-LacI exhibits a 20-fold weaker *in vivo* binding to *O*_sym_ compared to Wt-LacI (as shown by the Miller assay), yet maintains a similar association rate in single-molecule experiments *in vivo*, it follows that the difference in binding strength arises from changes in the dissociation rate. However, the ∼3-fold difference in *O*_sym_ dissociation rates between Q55N-LacI and Wt-LacI observed *in vitro* is insufficient to fully explain the 20-fold change in *in vivo O*_sym_ binding affinity, a discrepancy that is further discussed in the ‘Discussion’ section.

## Discussion

In this study, we investigated whether the relative stability of the search and recognition conformations of the TF LacI gives the predicted impact on search speed and operator binding strength, under the assumption that the evolutionary purpose of the conformation switching is to resolve the speed-stability paradox by having one conformation rapidly searching and the other conformation stably binding. Previous work identified the hinge region of LacI as critical for transitioning between the two conformations [28]. We hypothesized that mutations in the hinge region would alter the search or recognition conformation preference, thereby impacting binding stability to operator and nonoperator sites, and changing DNA specificity, with the associated impact on target search kinetics in the genome. Using MD simulations, we identified a LacI mutant: V52A-LacI, which has a hinge region that is more likely to occupy the helix conformation and thus presumably favours the recognition conformation in comparison with Wt-LacI. In addition, we identified Q55N-LacI using empirical predictions for short peptides [30]. This variant is predicted to favour the unstructured conformation, thus, presumably, is more likely to appear in the search conformation compared to Wt-LacI. *In vitro* DNA microarray experiments confirmed these predictions, with V52A-LacI showing stronger, but less specific binding and Q55N-LacI exhibiting higher specificity, but weaker binding.

For the Q55N-LacI mutant, its search time *in vivo* is similar to Wt. The time to access *O*_sym_ could, however, actually be shorter if the probability of binding *O*_sym_ after it has been reached is lower than Wt. In the *in vitro* experiment (Supplementary Fig. S1B) we find that this probability is decreased by 30% compared to Wt. If this is directly applicable in the *in vivo* situation this would imply a 30% decrease in the time to access to keep the search time the same as Wt. Q55N-LacI exhibited ∼20-fold weaker binding with *O_sym_* than Wt-LacI in the *in vivo* Miller assay (Fig. 3A), which is only partly explained by the three-fold faster dissociation observed *in vitro* (Fig. 2G, see Supplementary Table S5 for an integrated overview of comparative parameters across *in vitro* and *in vivo* experiments). The time for searching, which we can measure directly *in vivo* (Fig. 4), does not account for the remaining factor needed to get the low repression observed in the Miller assay. Clearly, some aspect of the *in vivo* situation is not accounted for. What could these missing aspects be?

A potential missing aspect could be differences in the ionic strengths used in the *in vitro* experiments and the *in vivo* situation inside living cells, where the sum of Na^+^ and K^+^ is 30 mM *in vitro* and 200–300 mM *in vivo*. However, EMSA equilibrium binding comparisons reveal that the Q55N-LacI affinity is less sensitive to changes in salt concentration as compared to Wt-LacI. Specifically, while both proteins show an increase in *K_D_* from 50 to 200 mM NaCl, the increase is substantially greater for Wt-LacI (∼10-fold) than for Q55N-LacI (∼2-fold) (Supplementary Fig. S7C and D). Based on the EMSA result we would expect the Q55N-LacI and Wt-LacI to become more similar going from *in vitro* to *in vivo* and not vice versa which is the observed. The lower sensitivity of the Q55N-LacI binding constant to increase in salt concentration as compared to Wt-LacI is qualitatively inline with a model where increasing salt concentrations primarily decreases the sliding length (as detailed in the SI).

Macromolecular crowding is present in the *in vivo* not in the *in vitro* experiments. Crowding could affect the *in vivo* situations in multiple ways: For bi-molecular interactions in 3D, obvious effects of crowding are increased concentration due to excluded volume and changes in diffusion rates. These effects will, however, most likely impact the three LacI variants equally. A more subtle effect of crowding is that it promotes compacted protein conformations in order to increase the entropy in the solvent [55], which may impact the LacI variants differently. Crowding would, to first approximation, promote the more compacted helix conformation. This would presumably make Q55N-LacI more similar to Wt *in vivo* as compared to *in vitro*, i.e. it cannot explain why Q55N-LacI binds worse *in vivo*. When there is crowding on DNA (i.e. 1D) the free sliding length is restricted by other DNA-binding proteins [56]. These reduced sliding lengths make the search slower, especially for proteins that slide far. On the other hand, the crowding also causes more return events to the operator after dissociation from the operator into the sliding mode. This leaves the equilibrium binding essentially unaltered and thus does not explain the difference between *in vitro* and *in vivo* affinities. Lastly, the crowding on DNA also decreases the amount of accessible nonspecific DNA and the fraction of time the operator is available, but these consequences are very similar for all variants. In summary, crowding cannot account for the differences between *in vitro* and *in vivo*.

There is a subtle difference between the strains used for the Miller assay (Fig. 3) and those used for the *in vivo* kinetics (Fig. 4). The strains used for the Miller assay, by necessity of the assay, have a promoter in front of the *O*_sym_ operator, while the strains used for *in vivo* kinetics do not. A reduced binding probability *in vivo*, as measured by the Miller assay, can, for example, be due to competition with RNAP (RNA polymerase) or active displacement of Q55N-LacI by RNAP. However, this possibility is ruled out by the close agreement between the ratio of *O*_sym_ occupancy between Q55N-LacI and Wt-LacI, obtained from the Miller assay (0.78) and single molecule experiments (0.59). This suggests that the presence of a promoter in front of *O*_sym_ does not dramatically decrease the overall *O_sym_* occupancy by Q55N-LacI compared to Wt-LacI in the Miller assay.

Finally, another out of equilibrium displacement mechanism could be due to chromosome supercoiling or dynamics. If this makes dissociation faster *in vivo* than on the short oligos presented in the *in vitro* experiment, this effect could be more pronounced for Q55N-LacI, which has a lower binding strength compared to Wt-LacI.

For V52A-LacI, experiments on living cells demonstrated that it repressed a reporter protein controlled by *O_sym_* stronger than Wt-LacI (Fig. 3A). The change in repression strength quantitatively corresponded to the slower dissociation rate for V52A-LacI *in vitro* (Fig. 2G), suggesting that its the search time for the *O*_sym_ site *in vivo* should be similar to V52A-LacI and Wt-LacI. At the same time, we observe a 4.8-fold increase in binding to nonoperator sequences in the absence of a specific operator for V52A-LacI as compared to Wt-LacI (Fig. 3D), which should increase the access time. At first glance, one could imagine that it is possible to argue that the expected increase in access time time, due to increased nonspecific interactions *in vivo*, could be counteracted by an increased probability of binding for V52A-LacI in order to keep the search time the same as for Wt-LacI. However, the suggestion that the search is similar for Wt-LacI and V52A-LacI rely on the assumption that the ratio of dissociation rates is the same *in vitro* and *in vivo*. Since also the dissociation rate depend on the binding probability [31], an argument that the binding probabilities changes differently for V52A-LacI as compared to Wt-LacI when transitioning from in *in vivo* and *in vitro*, would invalidate the underlying assumption that the dissociation rate ratio remains the same *in vivo* and *in vitro*. Possibly a more parsimonious interpretation is that the search time is indeed increased for V52A-LacI, due to the increased nonspecific interactions, and that the reason why this does not give rise to low operator binding strength *in vivo* is that the dissociation rate decreased by approximately the same amount.

Independent of what causes the discrepancy between the *in vitro* and *in vivo* observations, we find that Q55N-LacI represses poorly *in vivo*, although it associates with the operator as fast as Wt-LacI. This observation has important implications regarding our initial question, whether favoring the search conformation can make the association faster. It appears the answer is no; the interactions between Wt-LacI and nonoperator DNA sequences are already sufficiently low not to get trapped on nonoperator sequences for significant fractions of time.

If we instead tune LacI to favor the recognition conformation, we find that V52A-LacI enhances repression strength mainly by making dissociation slower, but at the same time, it loses inducibility. Taking into consideration that the V52A mutant has shown an IPTG affinity comparable to Wt-LacI [54], the likely explanation for the reduced inducibility is that V52A-LacI binds so stably to the *O_sym_* operator that it only rarely flips out of the specific bound conformation, limiting the opportunity for IPTG to bind V52A-LacI. Increased binding strength to the operator together with a reduced inducibility as compared to Wt-LacI has also previously been observed for the V52 mutants, V52A, V52H and V52S when binding the *O_1_* operator. However, Zhan *et al.*, 2006 reported that tetrameric V52 mutants did not enhance affinity for *O*_sym_, whereas our measurements with dimeric LacI variants measurements show a clear affinity increase for *O_sym_* both *in vitro* and *in vivo*. The divergence, likely rooted in oligomeric state or assay design, does not alter the qualitative message shared by both studies: hinge‐helix stabilization tightens DNA binding at the cost of IPTG responsiveness. We therefore attribute diminished inducibility to a shift of the conformational ensemble toward the recognition state, limiting access to the IPTG-accessible search state. This stability-inducibility trade-off is in line with earlier reports for mutations in other LacI domains [14, 57].

How do we reconcile that G58A-LacI displayed increased helix propensity as compared to Wt-LacI, based on theoretical predictions, and that it at the same time binds approximately as poorly as Q55N (Supplementary Fig. S8)? If switching between sliding and recognition confirmations is critical for facilitated diffusion, a TF that is locked to the recognition confirmation should bind very slowly. For example, the recognition conformation may require a bent DNA structure, thus limiting binding to, or sliding on, straight DNA. G58A may represent such a locked recognition state. What speaks against this idea is that G58A still has an association rate which is similar to wt-LacI on short oligos *in vitro* (Supplementary Fig. S8).

Circling back to our initial question, our data supports that the operator access time can be traded for bound complex stability, but that the association rate (search speed) is not increased by faster access since it also impacts the binding probability at the operator. Our observations instead favour the model in which the mechanistic challenge is to compromise between inducibility and repression strength. While it remains unclear whether Wt-LacI in its native context represents an evolutionary optimum with respect to this compromise, the existence of previously reported mutants [57, 58] with both enhanced repression and IPTG inducibility suggest that it is not. IPTG is, however, not LacI’s natural inducer and, therefore, it will be important to investigate whether these mutants keep enhanced repression and inducibility when interacting with the natural inducer (allolactose) in living cells to know if we still are missing pieces for understanding the evolutionary constraints shaping LacI function.

## Supplementary Material

gkag296_Supplemental_File

## Data Availability

The molecular dynamics simulation datasets, Miller assay data, EMSA gel Images, analysis scripts, and codes used in this study are available in the SciLifeLab Data Repository, https://doi.org/10.17044/scilifelab.29040599. Datasets of single-molecule live-cell imaging experiments and Protein Binding Microarray (PBM) assays are deposited in the BioImage Archive Repository, https://doi.org/10.6019/S-BIAD1940.
