# Supplementary material for: LacI strikes a balance between stability and inducibility
Source: Nucleic Acids Res. 2026 Apr 8;54(6):gkag296. doi: 10.1093/nar/gkag296 (PMC13069688; doi:10.1093/nar/gkag296)
Supplement: gkag296_Supplemental_File [file gkag296_supplemental_file.pdf]

Supplementary Text

Derive Microscopic binding rates from measured macroscopic rates (k_a, k_d) on PBM

To dissect the binding properties of the mutants at a more detailed level we adapted the theoretical approach from our previous study(1). The approach uses the macroscopic measurements k_a and k_d to many operators to make inferences about microscopic properties in special cases. In particular, if there is a linear anticorrelation between k_a and k_d then the operator binding is mainly governed by the probability, p_{tot}, of specifically binding when the TF is non-specifically bound on the operator, and less by the rate of switching back to search confirmation, $k_{off,u}$, when bound on the operator. For the V52A mutant, the anticorrelation trend is clear (Fig S1A), which makes it possible to infer its microscopic binding rates (see Material and Methods for details). Importantly, the microscopic rate of V52A for leaving the bound state is lower than Wt, which is consistent with that it prefers the helix conformation of the recognition complex. The probabilities for binding operators, p_{tot}, (Fig S1B) are also generally higher than for Wt For Q55N-LacI the specificity is so high that there is little data to apply the model to. This implies that p_{tot} are very low, except for O_{sym} which is still close to unity. For the operators where the binding and unbinding events are still possible to measure, there is an anticorrelation for k_a and k_d that suggests that the microscopic rate of switching back to the search confirmation is slightly higher than Wt-LacI (Fig S1D).

Salt dependence of K_D

How can we reconcile that the K_D of Q55N-LacI is less sensitive to ionic strength when compared to Wt-LacI? If we break down K_D into components, we can disentangle which factors may respond differently for mutant and Wt LacI.

$$K_D = \frac{k_{off,\mu}(1-p_{tot})}{k_{on,max}p_{tot}}$$ (Marklund et al. (1) Eq S18)

$k_{off,u}$ is different for the different mutants (Fig. S1), but since it is mainly due to factors inside the protein DNA complex, it is unlikely to change differentially with respect to salt concentration. $k_{on,max}$, the rate of a LacI protein entering into the testing state around the DNA region that contains O_{sym}, can potentially change differently for the different proteins, but it's not clear how. The impact of a change in ionic strength on p_{tot}, the probability of binding the operator before leaving that region of the DNA, is, however, easier to predict. To simplify the notation, we defined $(1-p_{tot})/p_{tot}$ $(=q)$, *i.e.,* a low q $(=1/p_{tot}-1)$, means a low K_D, which means a strong binding.

In a model where LacI slides in 1D on non-specific DNA before it macroscopically dissociates to an uncorrelated part of the chromosome, it returns to the same position on the DNA as many times as the number of base pairs in the sliding length(2). Thus, if we assume that the sliding length is N, p_{tot} is one minus the probability of failing to bind N consecutive attempts, *i.e* $p_{tot}=1-(1-p)^N$, where p is the probability of binding when the LacI is on top of the operator.

Based on previous studies, partly on other proteins, the ionic strength has a major effect on non-specific binding(3) and sliding length(4) but not diffusion rate(4). We can therefore evaluate what would be the effect on the binding constant, K_D, if we change the sliding length for proteins with different probabilities of binding when on top of the operator, p, assuming that p is not dependent on salt concentration. We can define the ratio of K_D as $Q=(1\text{-}p)^N/(1\text{-}(1\text{-}p)^N)\ /\ ((1\text{-}p)^n/(1\text{-}(1\text{-}p)^n))$, where N is the low salt sliding length (≈60) and n is the high salt sliding length (≈30). If we estimate p_{tot} from Fig S1b we get p_{tot}= 0.99 for WT and p_{tot} = 0.8 for Q55N, which translates to p=0.074 for WT and p=0.027 for Q55N, if we assume N=60 corresponding to the longer sliding in low salt. In terms of change to higher salt with shorter sliding, n=30, this gives Q=0.09 for WT and Q=0.31 for Q55N.

Supplementary Figures

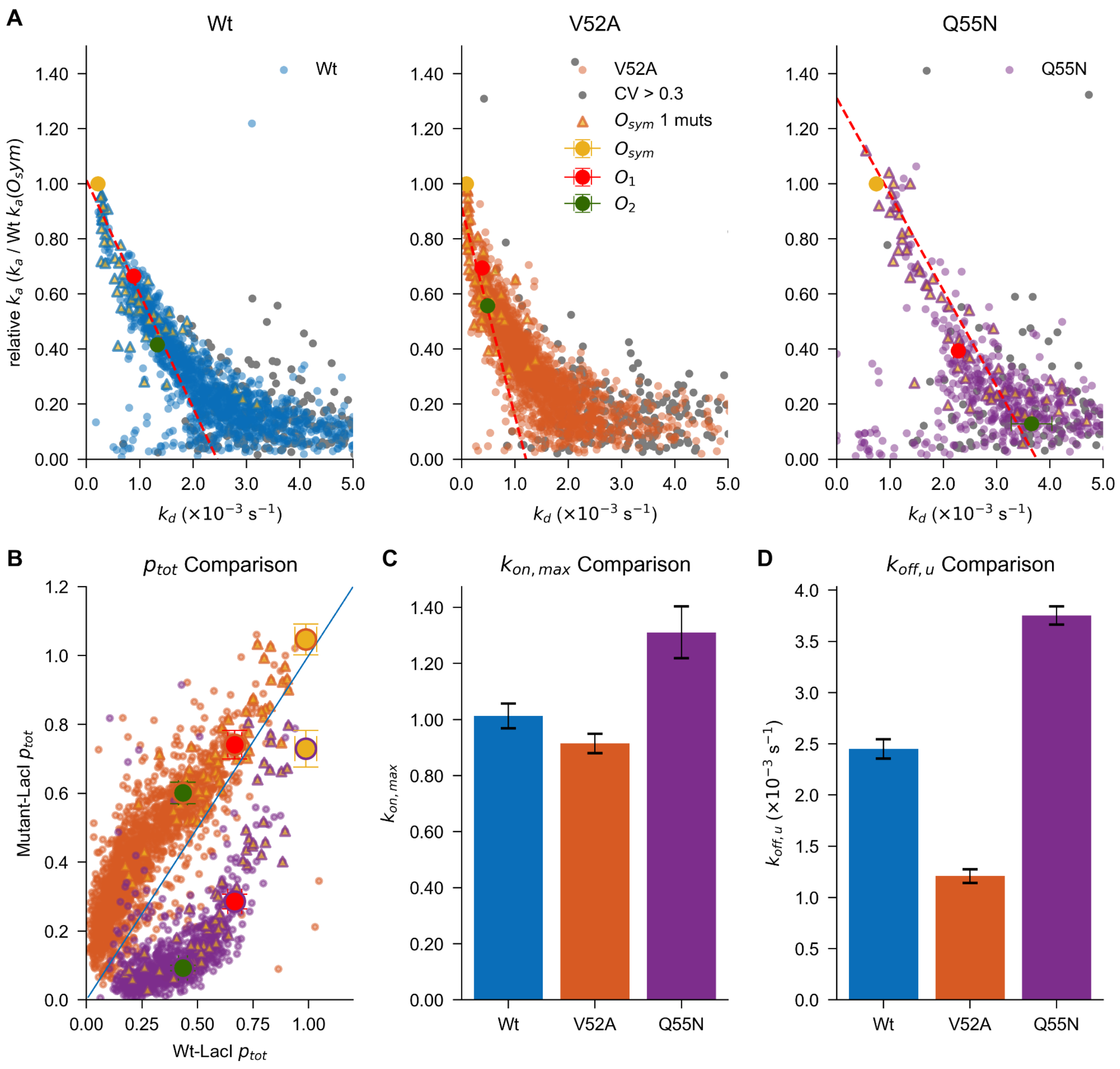

Figure S1. Measured k_a vs k_d rates for LacI variants binding with thousands DNA operators *in vitro*. A. Measured association and dissociation rates for LacI-Wt (Blue), LacI-V52A (Orange) and LacI-Q55N (Purple) binding with thousands of single and double mutants of three DNA operators of LacI (O_{sym}, O_1, O_2) *in vitro*. Each data point represents the mean k_a value for a unique DNA sequence, calculated from 10 replicates for Wt-LacI and 5 replicates for each mutant-LacI. Red dashed lines show the model prediction of $k_a = k_{on,max} - k_d * k_{on,max}/k_{off,\mu}$, where $k_{on,max}$ is the intercept of a linear fit to the top 35% of O_{sym} single mutants (CV < 0.3) and $k_{off,\mu}$ is the trimmed-mean microscopic unbinding rate estimated from all O_{sym}single mutants (see details in methods). Three LacI operators (O_{sym}, O_1, O_2) are labeled with yellow-, red-, and green-filled circles, as marked. Excluded sequences associated with weak binding or high variability (CV ≥ 0.3) of k_a, k_d measurement are marked in grey points as in Figure 2. Error bars on three operators are propagated standard error of mean (S.E.M.) of their fitted and normalized k_a and fitted k_d measurements. **B.** Calculated binding probability for Wt-LacI(X-axis) vs. mutants (Y-axis), according to Marklund et al.(1) for each protein (k_a / $k_{on,max}$), with y=x as blue line, representing Wt-LacI's p_{tot} for comparison. **C & D** The $k_{on,\ max}$ value is determined as the y-intercept from a linear fit performed on the top 35% of Osym single mutants, with its error bar representing the standard

error (SE). The mean and SEM for $k_{off,u}$ are calculated from a set of trimmed $k_{off,u}$ values—excluding the 10% most extreme measurements—from all obtained koff,u data. These $k_{off,u}$ values are computed by applying the equation $ka = kon,max - \frac{kon,max}{koff,u} \times kd$ (as presented in Eq. 1 of Marklund et al.(1)) to all valid Osym single mutants (depicted as triangles with yellow face colors in panel A) that have an estimated $k_{on,max}$ from the linear fit.

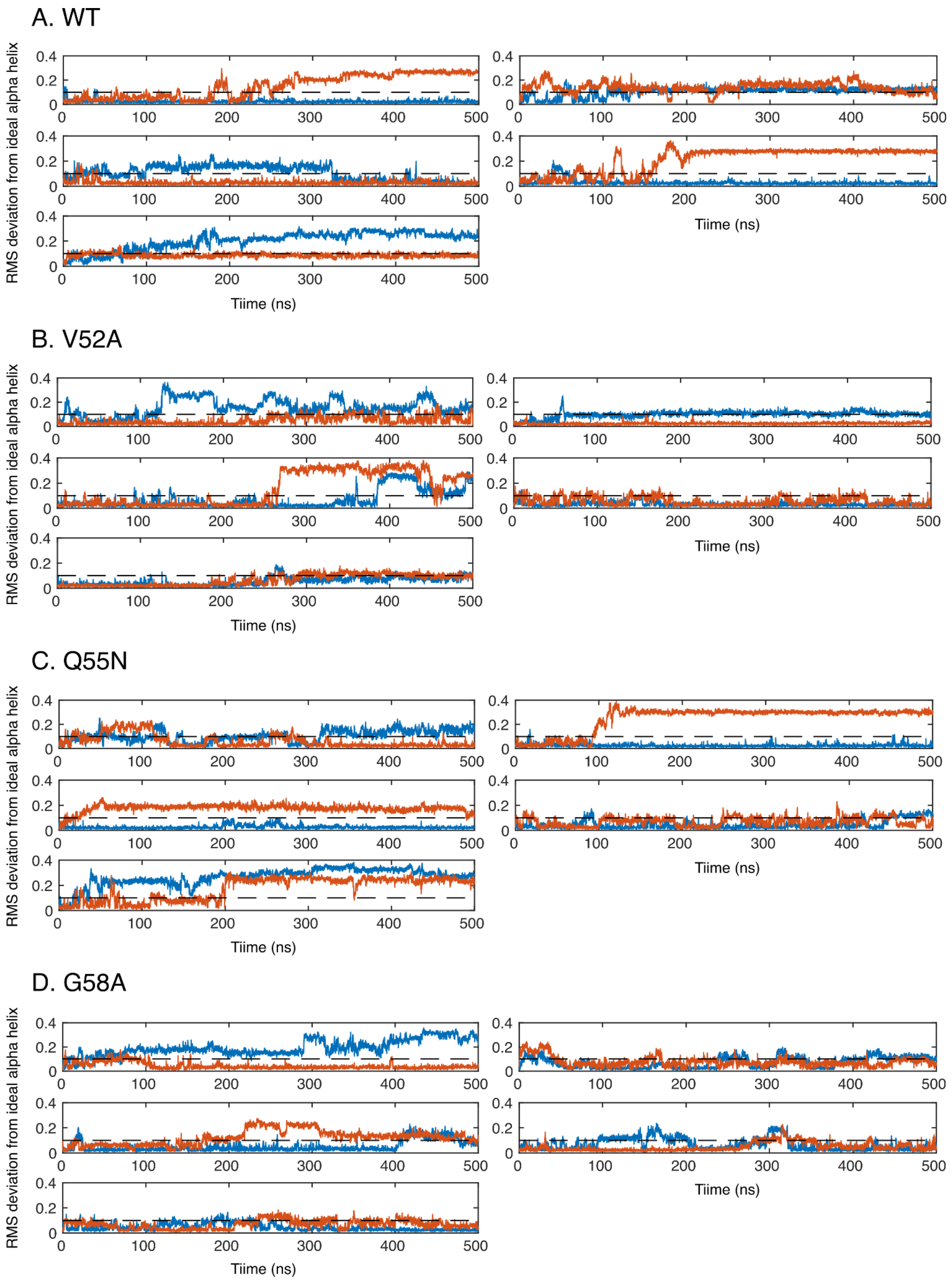

Figure S2: Root mean square deviation from ideal helix for residues R51-A57 as function of time for five replicate MD simulations of a LacI dimer. Each panel for each LacI variant contains results from one MD simulation. Red and blue lines show RMSDs for each of the two hinges in the LacI dimer. Black dashed line is RMSD=0.1nm, which is used as cut-off for assigning a hinge as helical in Fig. 1 of the main text.

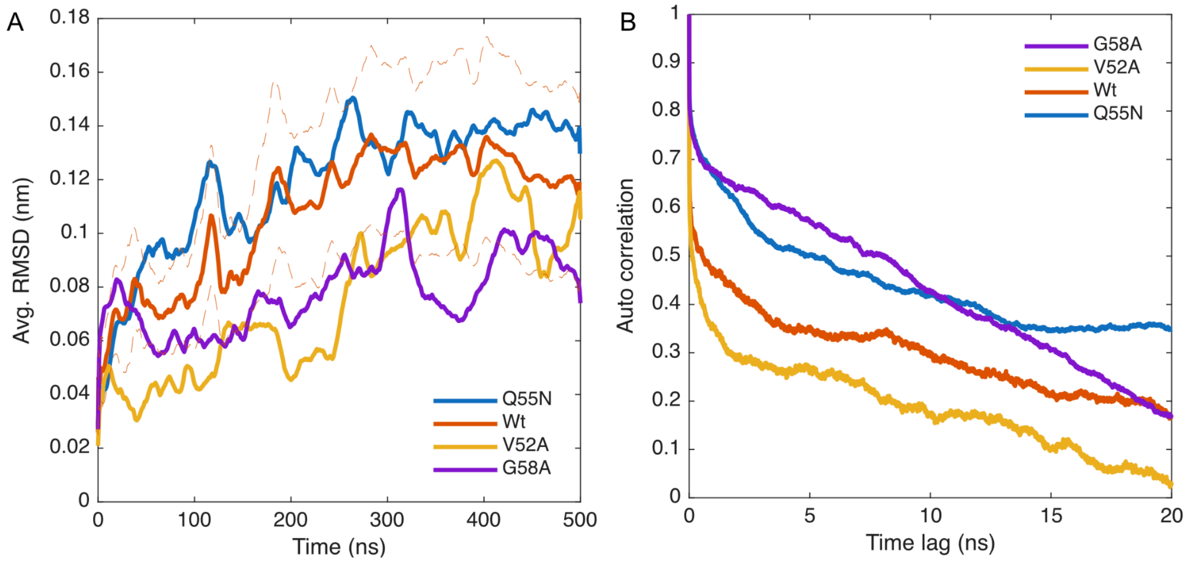

Figure S3: **A.** Solid lines are averages of the RMSD for all hinges for each LacI variant shown in Fig. S2. Curves are smoothed as in Fig. 2E of the main text. Dashed red lines show 1xSEM for each timepoint for the Wt case. The SEM curves are smoothed as in Fig. 2E. **B.** Auto correlations for time points before smoothing of the part of the time traces between 300ns and 500ns in Fig. 2E (left) of the main text.

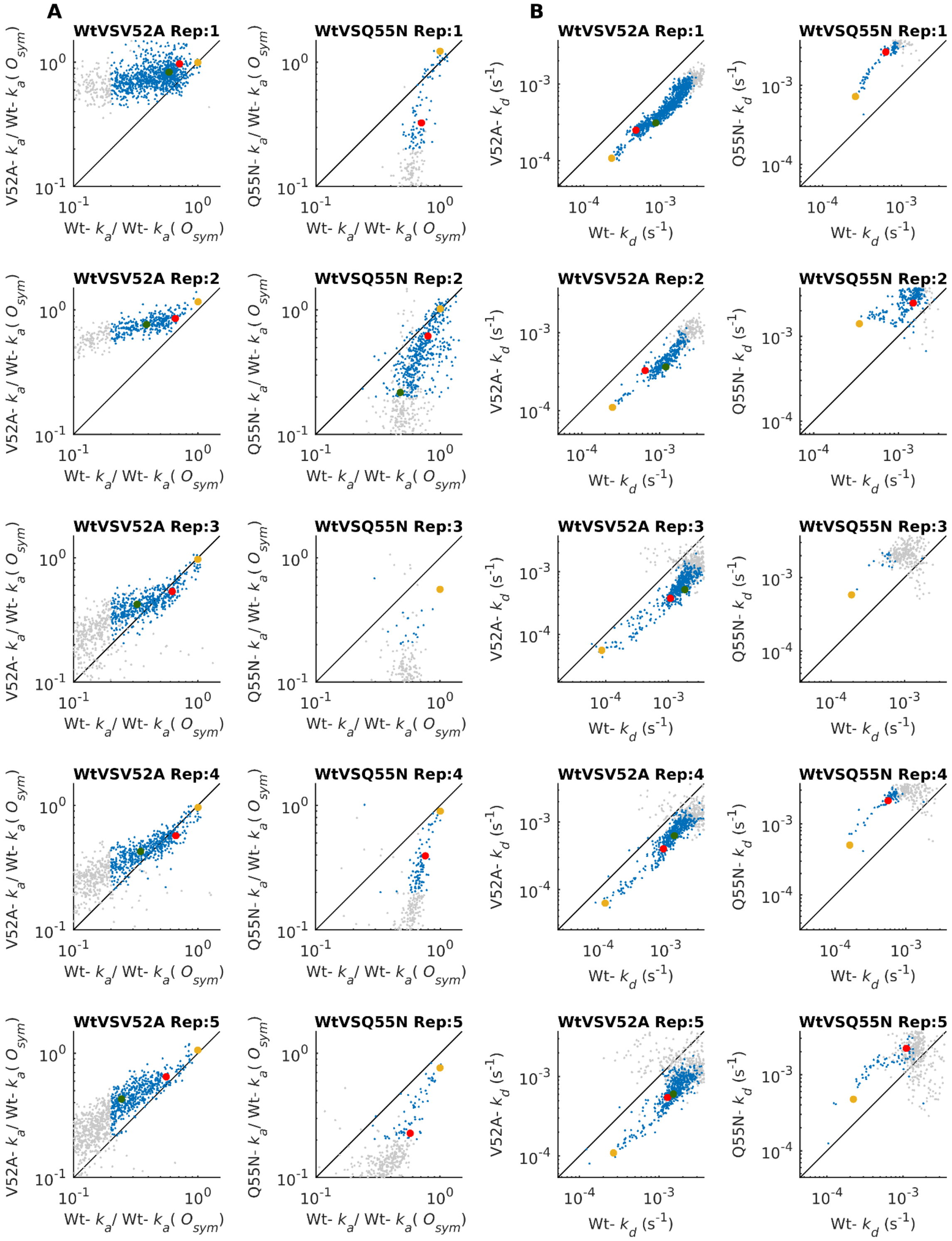
A
B
WtVSV52A Rep:1
WtVSQ55N Rep:1
WtVSV52A Rep:2
WtVSQ55N Rep:2
WtVSV52A Rep:3
WtVSQ55N Rep:3
WtVSV52A Rep:4
WtVSQ55N Rep:4
WtVSV52A Rep:5
WtVSQ55N Rep:5
V52A- k_a/ Wt- k_a(O_{sym})
Q55N- k_a/ Wt- k_a(O_{sym})
Wt- k_a/ Wt- k_a(O_{sym})
V52A- k_d (s^{-1})
Q55N- k_d (s^{-1})
Wt- k_d (s^{-1})

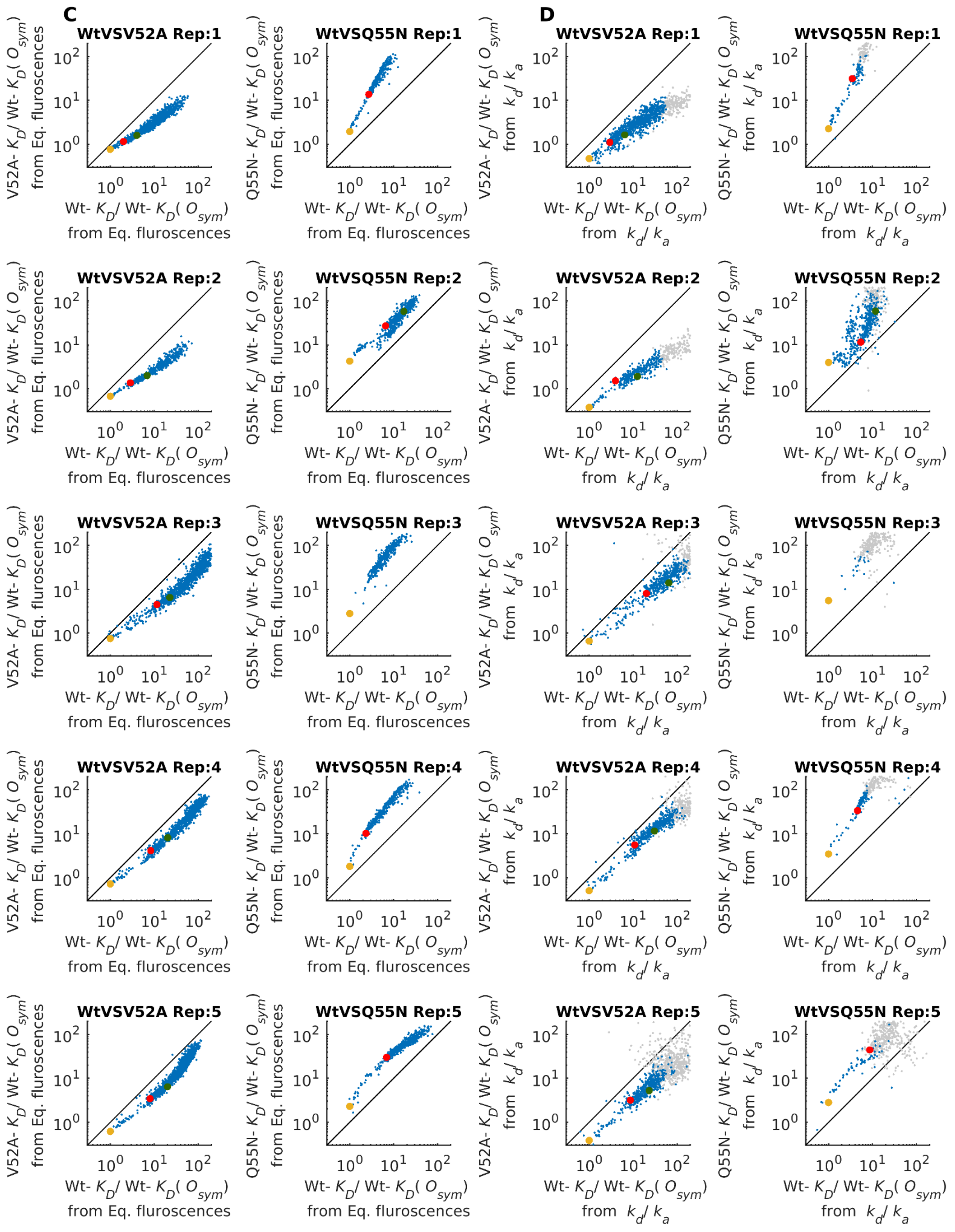

Figure S4 Individual Wt VS Mutant side by side experimental replicates for data shown in Figure 2C, D&E. In each replicate of the side-by-side PBM experiment, sequences with high k_a and k_d variability (CV > 1) or relative k_a (normalized to Wt- k_a (O_{sym}) within each replicate)<0.2 are shown in grey. K_D values calculated from k_a k_d measurements meeting CV > 1 or relative k_a <0.2 are also marked in grey.

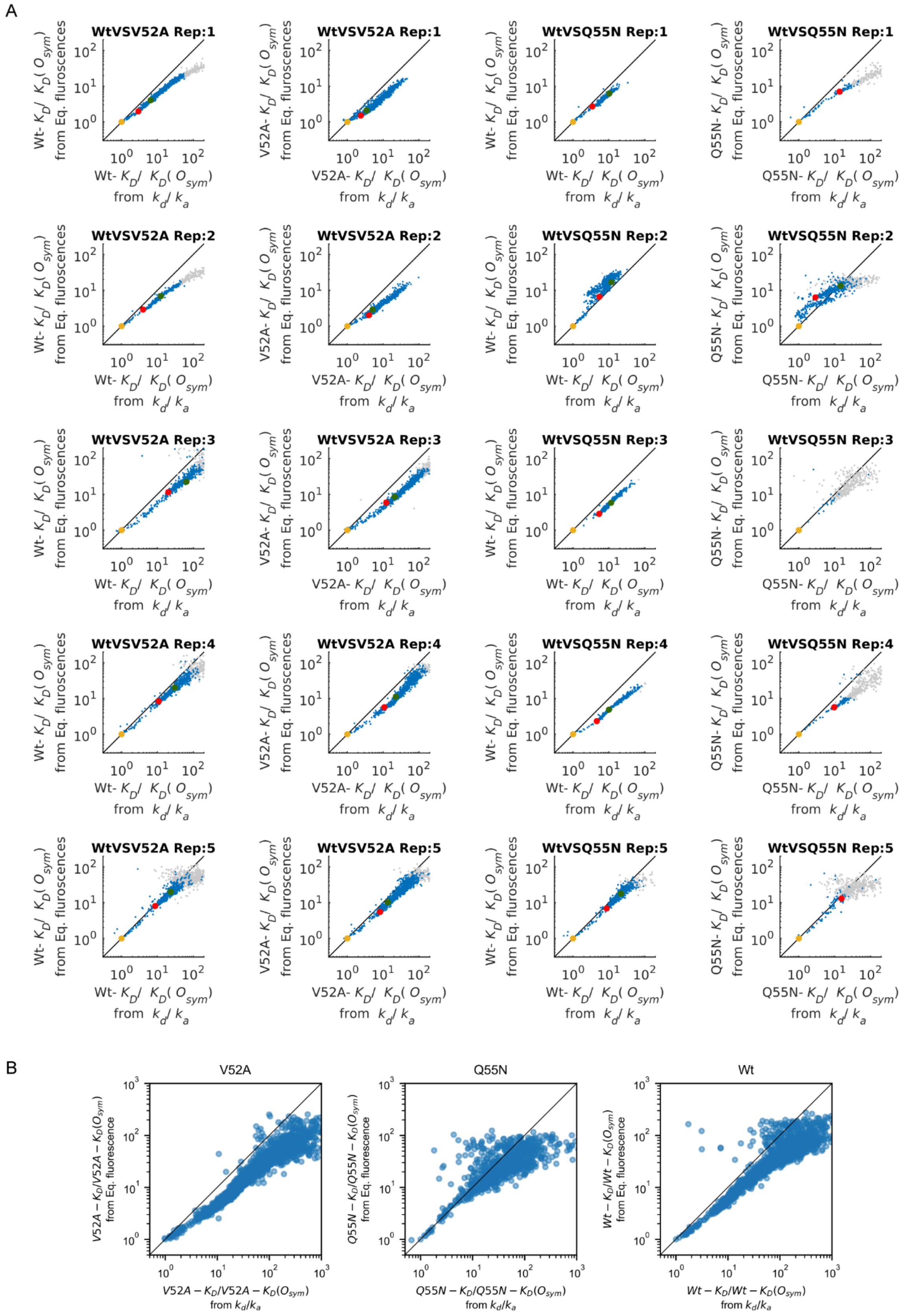

Figure S5 Correlation of relative K_d estimates from two different methods for calculating K_d. A. Individual correlation of K_d estimates from two methods for each protein in each

experiment. **B.** Correlation of mean K_d estimates from two methods, each data point of K_d is taken from the mean value of 10 replicates for Wt, 5 replicates for V52A and 5 replicates for Q55N.

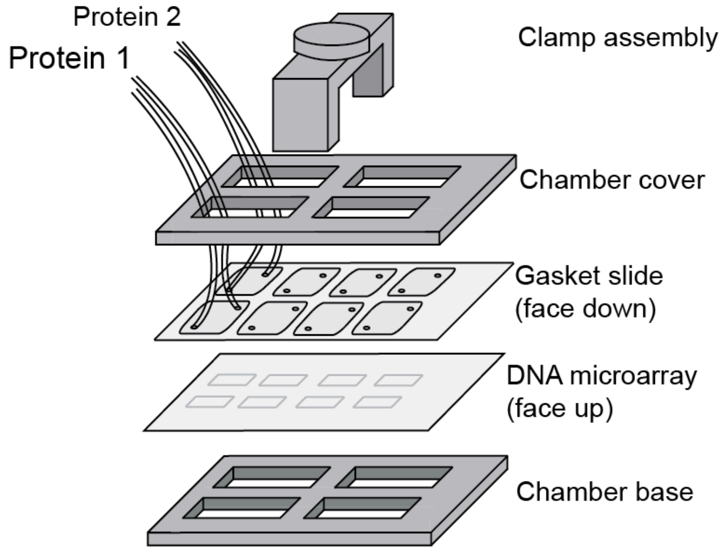

Figure S6 Device set up for measuring kinetics in vitro with Protein binding Microarrays

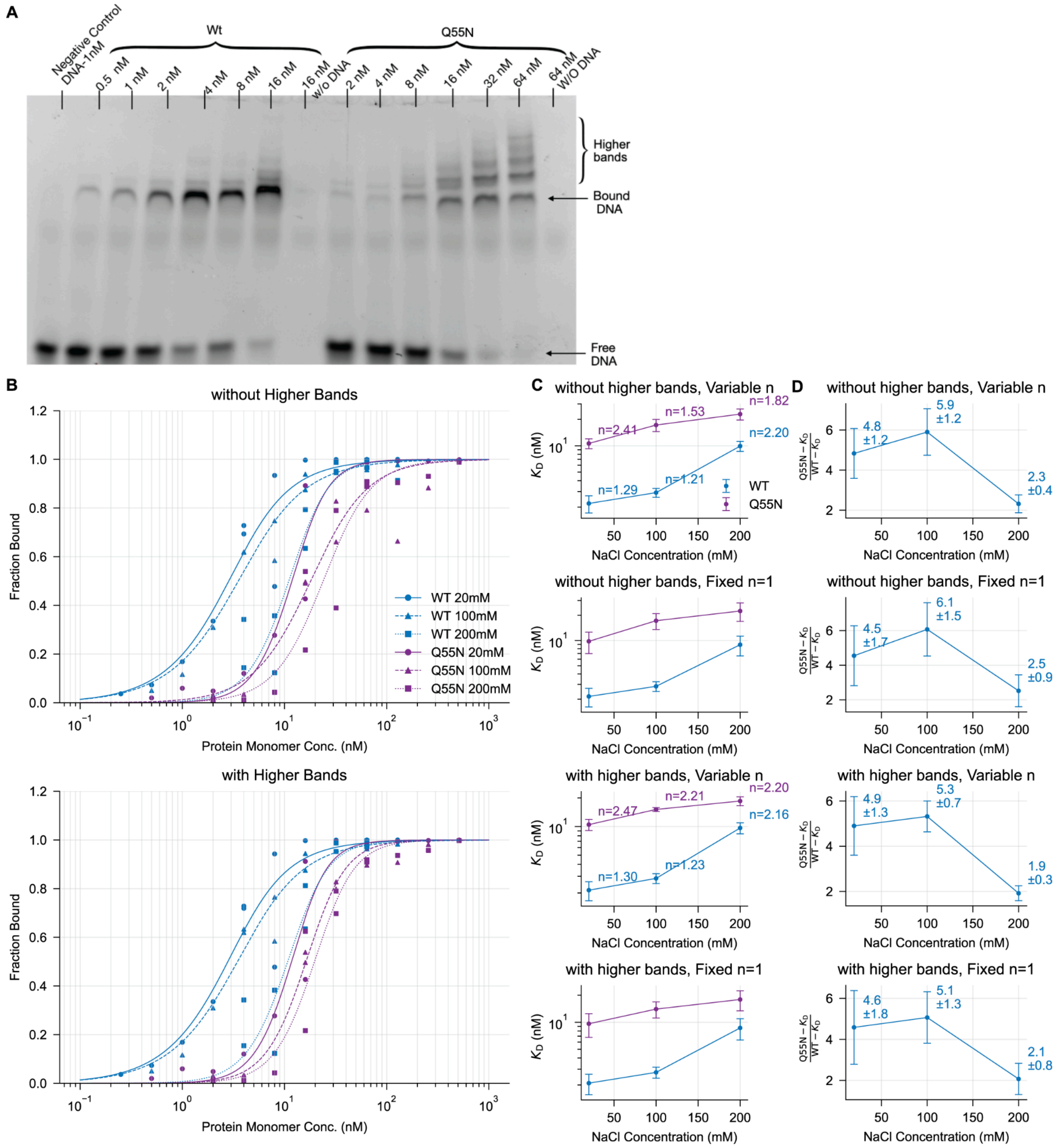

Figure S7 EMSA-salt titration for Wt-LacI and Q55N-LacI **A**. Example of Electrophoretic Mobility Shift Assay (EMSA) gel showing the binding of wild-type (WT) and Q55N mutant LacI to DNA (a 60bp DNA fragment including O_{sym}) at six different protein monomer concentrations in a 100 mM salt condition. The higher bands of DNA, LacI-bound DNA and free DNA are indicated in the image. note: LacI concentration labelled here are all for LacI monomers. **B**. Binding curves for the fraction of LacI-bound DNA were fitted using two models: a simple binding model with a fixed Hill coefficient (n=1) and a Hill model with a variable Hill coefficient (n). In the simple binding model, the fraction of LacI-bound DNA, f, is described by $f(L, Kd, D) = \frac{(L+D+Kd)-\sqrt{(L+D+Kd)^2-4LD}}{2D}$ calculates the fraction of DNA bound by solving the quadratic equation for the equilibrium between protein (L, denoted in equation for total protein concentration), DNA (D, denoted in equation for total DNA concentration), and the dissociation constant (Kd). The Hill model, solves a system of two equations incorporating the Hill equation $\theta = \frac{Lfree^n}{Kd^n+Lfree^n}$ and the conservation of mass $L_{free} = L_{total} -$

$n*D*\theta$, where θ is the fraction of DNA bound, L_{free} is the free protein concentration, L_{total} is the total protein concentration, and n is the Hill coefficient. The system is solved numerically for each protein tested to determine its *Kd* and Hill coefficient in three salt concentrations. The best-fit model was selected based on the lowest residual sum of squares and then is plotted. **C.** Equilibrium dissociation constants (K_d) determined from B for Wt-LacI and Q55N-LacI at varying NaCl concentrations. Data are shown as K_d values with their corresponding standard errors, comparing salt-dependent inverse of affinities across four distinct analysis scenarios **D**. Corresponding ratios of Q55N-LacI Kd to Wt-LacI Kd at each salt concentration, illustrating relative changes in salt sensitivity. Error bars represent propagated standard errors from panel C.

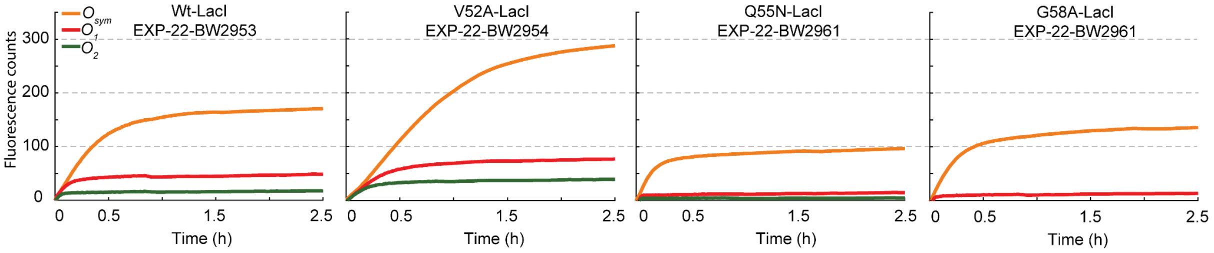

Figure S8 Initial screening: Binding curves for four LacI variants (WT, V52A, G55N, G58A) to O_{sym} (orange), O_1 (red), and O_2 (green) operators. Plots display fluorescence counts over time with the negative control signal subtracted.

Supplementary Tables:

Table S1. Bacteria strains used in this study

**Strains**	**Genotype or Relevant characteristics[1]**	**Reference or source**
**Microscopy (single molecule experiment)**		
EL8	*Source for selectable/counterselectable marker, Acatsac1*	Gift from Dan I. Andersson lab (DA58376)
EL2620	Δ*fliA*::Kan^R *(Source for kanamycin* resistance cassette)	Strain associated to (5)
EL3498	*lacI-mVenus* Δ(p_{lac}-*lacZ*)::term	JE107 (6) with phi80 removed<br>This work
EL3518	*lacI(V52A)-mVenus* Δ(p_{lac}-*lacZ*)::term	This work
EL3520	*lacI(Q55N)-mVenus* Δ(p_{lac}-*lacZ*)::term	This work
EL4053	*lacI-mVenus* Δ(p_{lac}-*lacZ*)::term *aslA-glmZ* intergenic::cat- lacOsym	This work
EL4089	*lacI-mVenus* Δ(p_{lac}-*lacZ*)::term *aslA-glmZ* intergenic::cat-O_{sym}-*lacZ*	This work
EL4210	Kan^R-p_{FAB138}-*lacI-mVenus* Δ(p_{lac}-*lacZ*)::term	This work
EL4219	Kan^{R}-p_{FAB138}-*lacI-mVenus* Δ(p_{lac}-*lacZ*)::term	This work
EL4226	Kan^R-p_{FAB138}-*lacI-venus* Δ(p_{lac}-*lacZ*)::term aslA-glmZ intergenic::cat-lacOsym	This work
EL4242	*lacI(V52A)-mVenus* Δ(p_{lac}-*lacZ*)::term *aslA-glmZ* intergenic::cat- lacOsym	This work
EL4243	*lacI(Q55N)-mVenus* Δ(p_{lac}-*lacZ*)::term *aslA-glmZ* intergenic::cat- lacOsym	This work
EL4264	Kan^R-p_{FAB138}-*lacI(V52A)-mVenus* Δ(p_{lac}-*lacZ*)::term	This work
EL4265	Kan^R-p_{FAB138}-*lacI(Q55N)-mVenus* Δ(p_{lac}-*lacZ*)::term	This work
EL4266	Kan^R-p_{FAB138}-*lacI(V52A)*-mVenus Δ(p_{lac}-*lacZ*)::term	This work

EL4267	Kan^R-p_{FAB138}-*lacI(Q55N)-mVenus* Δ(p_{lac}-*lacZ*)::term	This work
EL4268	Kan^R-p_{FAB138}-*lacI(V52A)-mVenus* Δ(p_{lac}-*lacZ*)::term *aslA-glmZ* intergenic::cat-lacOsym	This work
EL4269	Kan^R-p_{FAB138}-*lacI(V52A)-mVenus* Δ(p_{lac}-*lacZ*)::term *aslA-glmZ* intergenic::cat-lacOsym	This work
**Strains for Miller assay**		
EL409	*lacI-mVenus O1-* lacOsym *O2-*	This work
EL4491	Kan^R-p_{FAB138}-*lacI-mVenus* Δ(p_{lac}-*lacZ*)::term *aslA-glmZ* intergenic::cat-lacOsym-*lacZ*	This work
EL4493	Kan^R-p_{FAB138}-*lacI(V52A)-mVenus* Δ(p_{lac}-*lacZ*)::term *aslA-glmZ* intergenic::cat-lacOsym-*lacZ*	This work
EL4495	Kan^R-p_{FAB138}-*lacI(Q55N)-mVenus* Δ(p_{lac}-*lacZ*)::term *aslA-glmZ* intergenic::cat-lacOsym-*lacZ*	This work
EL4536	Kan^R-p_{FAB138}-*mVenus Δ(lacI-lacZ)*::term	This work
EL4580	Kan^R-p_{FAB138}-*mVenus Δ(lacI-lacZ)*::term *aslA-glmZ*::cat-lacOsym-*lacZ*	This work
EL4621	*Δ(mhpR-lacZ)*::catsacB	This work
EL4630	Kan^R-p_{FAB138}-*lacI-halo Δ(mhpR-lacZ)*	This work
EL4632	Kan^R-p_{FAB138}-*lacI*(V52A)-*halo Δ(mhpR-lacZ)*	This work
EL4634	Kan^R-p_{FAB138}-*lacI*(Q55N)-*halo Δ(mhpR-lacZ)*	This work
EL4639	Kan^R-p_{FAB138}-*lacI-halo Δ(mhpR-lacZ) aslA-glmZ* intergenic::cat-lacOsym-*lacZ*	This work
EL4641	Kan^R-p_{FAB138}-*lacI*(V52A)-*halo Δ(mhpR-lacZ)* *aslA-glmZ* intergenic::cat-lacOsym-*lacZ*	This work
EL4643	Kan^R-p_{FAB138}-*lacI*(Q55N)-*halo Δ(mhpR-lacZ)* *aslA-glmZ* intergenic::cat-lacOsym-*lacZ*	This work

[1]cat, gene encoding for CAT (Chloramphenicol acetyl-transferase)

Table S2. Primers used in this study

Primers	Sequence (5'3')[1]	Comments
aslA-hom-cat-Osym-Fwd	aggagagcgttttcaatcctacctctggcgcagttgatatTTACGCCCCGCCCTGCCAC	Primer to obtain fragment cat-lacOsym or cat-lacO_{sym}-lacZ fragment; Forward primer
aslA-hom-cat-Osym-Rev	agcatcaataatcaacgcgatataataaacctgccttacaattgtgagcgctcacaattCACGTAAGAGGTTCCAACTTTCACC	Primer to obtain cat-lacOsym fragment; Reverse primer
cat-Osym-lacZ_aslA_R	agcatcaataatcaacgcgatataataaacctgccttacTTATTTTTGACACCAGACCAACTGG	Primer to obtain cat-lacO_{sym}-lacZ fragment; Reverse primer
Comp2_lacI 14 bp_RBS_p138-Fw	gttactggtttcacattcaccaccctgaattgactctcttatacgttaaatctacgagccggatgattaattgtctaacaccgtgCGCAAGCGCAAAGAGAAAGC	Primer to obtain Kan^R-pFAB138-RBS-lacI(14bp) fragment; Forward primer
Comp1_Kan-Rv	gtgcaccaggtgcaccacgttgttttaactatagaaatgTCAGAAGAACTCGTCAAGAAGG	Primer to obtain Kan^R-pFAB138-RBS-lacI(14bp) fragment; Reverse primer

LacI_Halo_R	cctattctctagaaagtatagggcaattctcgagcacaagTTATTAACCGTGATGGTGATGG	Primer to obtain fragments, KanR-pFAB138-lacI-halo or KanR-pFAB138-lacI(V52A)-halo or KanR-pFAB138-lacI(Q55N)-halo; Reverse
insert aslA-glmZ_fw	GATAATTGAGATCCCTCTCCC	Sequencing primers to verify insert at aslA-glmZ intergenic region
insert aslA-glmZ_rv	ACCTGAGCTTGATCCTACAC	Sequencing primers to verify insert at aslA-glmZ intergenic region
lacI insert_mhpA_fw	GGTTAACAGCAGGCTGGATGTC	Sequencing primer to verify *lacI-mVenus*
kan_insert_seq	CAACCTTACCAGAGGGCGCC	Sequencing primer to verify KanR-pFAB138-*lacI-mVenus*
venus-lacY-DN-conf - rv	CCACAGCAGGTATTTGCGCAGC	Sequencing primer to verify *lacI-mVenus*
CAT-R	GCAACTGACTGAAATGCCTC	Sequencing primer to verify cat-lacOsym or cat-lacO_{sym}-lacZ insert
T7 Term_fwd	CTAGCATAACCCCTTGGGGC	Sequencing primer to verify *lacI-halo* insert
Seq_Halo_F	AGTTCATCCGCCCTATCCC	Sequencing primer to verify *lacI-halo* insert
Seq_Halo_R	CTTCGACCAGCGCGACGATG	Sequencing primer to verify *lacI-halo* insert

lacI mid rev3252	ACGCGCCGAGACAGAA CTTA	Sequencing primer *lacI-halo* insert

[1]Nucleotide sequences in lowercase are the 40 bases homology for lambda red

Table S3. Amino acid sequences of LacI-Halo variants for Protein binding Microarray assay

The Halotag used for fluorescent labelling, which was introduced in the C terminal of the LacI sequence is underlined. The linker sequence between the Halotag and the original LacI sequence is marked in bold. Mutations for LacI are marked in color. 6x-His tags were introduced for protein purification.

Name	Sequence
LacI-Halo	MKPVTLYDVAEYAGVSYQTVSRVVNQASHVSAKTREKVEAAMAEL<br>NYIPNRVAQQLAGKQSLLIGVATSSLALHAPSQIVAAIKSRADQLGAS<br>VVVSMVERSGVEAAKAAVHNLLAQRVSGLIINYPLDDQDAIAVEAA<br>ATNVPALFLDVSDQTPINSIIFSHEDGTRLGVEHLVALGHQQIALLAGP<br>LSSVSARLRLAGWHKYLTRNQIQPIAEREGDWSAMSGFQQTMQML<br>NEGIVPTAMLVANDQMALGAMRAITESGLRVGADISVVGYDDTEDS<br>SCYIPPLTTIKQDFRLLGQTSVDRLLQLSQGQCVKGNQLLPVSLVKRK<br>TTLAPNTQT**G**AEIGTGFPFDPHYVEVLGERMHYVDVGPRDGTPVLF<br>LHGNPTSSYVWRNIIPHVAPTHRCIAPDLIGMGKSDKPDLGYFFDDH<br>VRFMDAFIEALGLEEVVLVIHDWGSALGFHWAKRNPERVKGIAFME<br>FIRPIPTWDEWPEFARETFQAFRTTDVGRKLIIDQNVFIEGTLPMGVV<br>RPLTEVEMDHYREPFLNPVDREPLWRFPNELPIAGEPANIVALVEEYM<br>DWLHQSPVPKLLFWGTPGVLIPPAEAARLAKSLPNCKAVDIGPGLNL<br>LQEDNPDLIGSEIARWLSTLEISGHHHHHHG
V52ALacI-Halo	MKPVTLYDVAEYAGVSYQTVSRVVNQASHVSAKTREKVEAAMAEL<br>NYIPNRAAQQLAGKQSLLIGVATSSLALHAPSQIVAAIKSRADQLGAS<br>VVVSMVERSGVEAAKAAVHNLLAQRVSGLIINYPLDDQDAIAVEAA<br>ATNVPALFLDVSDQTPINSIIFSHEDGTRLGVEHLVALGHQQIALLAGP<br>LSSVSARLRLAGWHKYLTRNQIQPIAEREGDWSAMSGFQQTMQML<br>NEGIVPTAMLVANDQMALGAMRAITESGLRVGADISVVGYDDTEDS<br>SCYIPPLTTIKQDFRLLGQTSVDRLLQLSQGQCVKGNQLLPVSLVKRK<br>TTLAPNTQT**G**AEIGTGFPFDPHYVEVLGERMHYVDVGPRDGTPVLF<br>LHGNPTSSYVWRNIIPHVAPTHRCIAPDLIGMGKSDKPDLGYFFDDH<br>VRFMDAFIEALGLEEVVLVIHDWGSALGFHWAKRNPERVKGIAFME<br>FIRPIPTWDEWPEFARETFQAFRTTDVGRKLIIDQNVFIEGTLPMGVV<br>RPLTEVEMDHYREPFLNPVDREPLWRFPNELPIAGEPANIVALVEEYM<br>DWLHQSPVPKLLFWGTPGVLIPPAEAARLAKSLPNCKAVDIGPGLNL<br>LQEDNPDLIGSEIARWLSTLEISGHHHHHHG
Q55NLacI-Halo	MKPVTLYDVAEYAGVSYQTVSRVVNQASHVSAKTREKVEAAMAEL<br>NYIPNRVAQNLAGKQSLLIGVATSSLALHAPSQIVAAIKSRADQLGAS<br>VVVSMVERSGVEAAKAAVHNLLAQRVSGLIINYPLDDQDAIAVEAA<br>ATNVPALFLDVSDQTPINSIIFSHEDGTRLGVEHLVALGHQQIALLAGP<br>LSSVSARLRLAGWHKYLTRNQIQPIAEREGDWSAMSGFQQTMQML<br>NEGIVPTAMLVANDQMALGAMRAITESGLRVGADISVVGYDDTEDS<br>SCYIPPLTTIKQDFRLLGQTSVDRLLQLSQGQCVKGNQLLPVSLVKRK<br>TTLAPNTQT**G**AEIGTGFPFDPHYVEVLGERMHYVDVGPRDGTPVLF<br>LHGNPTSSYVWRNIIPHVAPTHRCIAPDLIGMGKSDKPDLGYFFDDH<br>VRFMDAFIEALGLEEVVLVIHDWGSALGFHWAKRNPERVKGIAFME<br>FIRPIPTWDEWPEFARETFQAFRTTDVGRKLIIDQNVFIEGTLPMGVV

	RPLTEVEMDHYREPFLNPVDREPLWRFPNELPIAGEPANIVALVEEYM<br>DWLHQSPVPKLLFWGTPGVLIPPAEAARLAKSLPNCKAVDIGPGLNL<br>LQEDNPDLIGSEIARWLSTLEISGHHHHHHG

Table S4. Occurrences of Operator Binding Sites in the *E. coli* Genome

The table lists the counts of operator mutation types located within the *E. coli* chromosome, with their relative positions to *OriC* indicated in brackets for mutations occurring fewer than 3 times.

Mutation Category	Osym	O1	O2	Total
No mutation	1(-61071)	0	0	1
Single Mutation	0	0	0	0
Double Mutations	0	0	0	0
Triple mutations	0	1 (+820407)	1 (+2844762)	4
Quadruple mutations	14	12	14	66
Total	15	13	15	71

Table S5. Comparative parameters of Wt-LacI and hinge-helix mutants (V52A, Q55N) from *in vitro* and *in vivo* assays

Expriment Type	LacO Type	LacI-Halo variant	k_a/ Wt-$k_a(O_{sym})$	k_d (±SEM) $\times 10^{-4}$ s^{-1}	K_D/ Wt-$K_D(O_{sym})$			
***In Vitro*: HT Kinetics Measurement on PBM**	O_{sym}	**Wt**	1 (ref.)	2.14 ± 0.03	1 (ref.)			
		**V52A**	1.03 ± 0.03	0.89 ± 0.02	0.4 (affinity ↑)			
		**Q55N**	0.89 ± 0.03	7.39 ± 0.13	3.6 (affinity ↓)			
	O_1	**Wt**	0.66 ± 0.02	8.84 ± 0.12	6.1			
		**V52A**	0.72 ± 0.03	3.80 ± 0.06	2.6			
		**Q55N**	0.35 ± 0.01	22.79 ± 0.89	28.4			
	O_2	**Wt**	0.42 ± 0.01	13.25 ± 0.35	14.1			
		**V52A**	0.58 ± 0.02	4.84 ± 0.10	4.1			
		**Q55N**	0.12 ± 0.01	36.53 ± 3.80	140.5			
/	/	**LacI-mVenus Variant**	/	/	**- IPTG**		**+ IPTG**	
					ϕ^{1}	$p_{bound}{}^{2}$	ϕ^{1}	$p_{bound}{}^{2}$
***In vivo*: Miller assay**	O_{sym}	**Wt**	/	/	38.9	97%	1.3	23%
		**V52A**	/	/	57.7	98%	8.8	89%
		**Q55N**	/	/	4.2	76%	1	0%
		**ΔLacI**	/	/	1 (ref.)	/	1 (ref.)	/
/			$k_a{}^{3}$ min^{-1}	/	**Δavg.N(dots)/cell[4]:**		**Δavg.N(dots)/cell[4]:**	
***In vivo*: Single-molecule assay**	O_{sym}	**Wt**	1.3	/	0.34 ± 0.02		0.04 ± 0.01	
		**V52A**	/	/	0.28 ± 0.02		0.33 ± 0.02	
		**Q55N**	1.3	/	0.20 ± 0.01		0.00 ± 0.00	

[1] ϕ: Repression strength, denoted detaily in the main text.

[2] p_{bound}: O_{sym} occupancy, see derivation in **Methods**.

[3] k_a: Fitted k_a values, taken from Figure 4C

[4]**Δavg.N(dots)/cell**: Taken from Figure 3E.

Table S6: Lower bounds of distance restraints between alpha carbons that were used in MD simulation.

DBD on Chain A		DBD on Chain B	
Residue pair	Lower bound (nm)	Residue pair	Lower bound(nm)
Thr34-Asp88	1.5	Thr34-Asp88	2.0
Thr34-Ala106	1.8	Thr34-Ala106	2.2
Thr34-Ala110	2.0	Thr34-Ala110	2.0
Glu39-Asp88	1.4	Glu39-Asp88	1.5
Glu39-Ala106	1.4	Glu39-Ala106	1.5
Glu39-Ala110	1.3	Glu39-Ala110	1.8
Ala43-Ala106	1.3	Ala43-Ala106	1.4
Ala43-Ala110	1.3	Ala43-Ala110	1.3

Reference

1. Marklund,E., Mao,G., Yuan,J., Zikrin,S., Abdurakhmanov,E., Deindl,S. and Elf,J. (2022) Sequence specificity in DNA binding is mainly governed by association. *Science*, **375**, 442–445.

2. Berg,O.G., Mahmutovic,A., Marklund,E. and Elf,J. (2016) The helical structure of DNA facilitates binding. *J. Phys. A: Math. Theor.*, **49**, 364002.

3. Wang,A.C., Revzin,A., Butler,A.P. and von Hippel,P.H. (1977) Binding of E.coli lac repressor to non-operator DNA. *Nucleic Acids Res.*, **4**, 1579–1593.

4. Blainey,P.C., van Oijen,A.M., Banerjee,A., Verdine,G.L. and Xie,X.S. (2006) A base-excision DNA-repair protein finds intrahelical lesion bases by fast sliding in contact with DNA. *Proc. Natl. Acad. Sci. U. S. A.*, **103**, 5752–5757.

5. Karempudi,P., Amselem,E., Jones,D., Khaji,Z., Tenje,M. and Elf,J. (2023) Real-time pooled optical screening with single-cell isolation capability. *bioRxiv, 558600v1*.

6. Hammar,P., Leroy,P., Mahmutovic,A., Marklund,E.G., Berg,O.G. and Elf,J. (2012) The lac repressor displays facilitated diffusion in living cells. *Science*, **336**, 1595–1598.
